# A Bayesian modelling framework with model comparison for epidemics with super-spreading

**DOI:** 10.1016/j.idm.2025.07.017

**Published:** 2025-08-05

**Authors:** Hannah Craddock, Simon E.F. Spencer, Xavier Didelot

**Affiliations:** a Department of Statistics, University of Warwick, United Kingdom; b Qualcomm Institute, University of California San Diego, San Diego, CA, USA; c School of Life Sciences, University of Warwick, United Kingdom

**Keywords:** Infectious disease epidemiology, Bayesian modelling, Model comparison, Super-spreading, Transmission heterogenity

## Abstract

The transmission dynamics of an epidemic are rarely homogeneous. Super-spreading events and super-spreading individuals are two types of heterogeneous transmissibility. Inference of super-spreading is commonly carried out on secondary case data, the expected distribution of which is known as the offspring distribution. However, this data is seldom available. Here we introduce a multi-model framework fit to incidence time-series, data that is much more readily available. The framework consists of five discrete-time, stochastic, branching-process models of epidemics spread through a susceptible population. The framework includes a baseline model of homogeneous transmission, a unimodal and a bimodal model for super-spreading events, as well as a unimodal and a bimodal model for super-spreading individuals. Bayesian statistics is used to infer model parameters using Markov Chain Monte-Carlo methods. Model comparison is conducted by computing Bayes factors, with importance sampling used to estimate the marginal likelihood of each model. This estimator is selected for its consistency and lower variance compared to alternatives. Application to simulated data from each model identifies the correct model for the majority of simulations and accurately infers the true parameters, such as the basic reproduction number. We also apply our methods to incidence data from the 2003 SARS outbreak and the Covid-19 pandemic caused by SARS-CoV-2. Model selection consistently identifies the same model and mechanism for a given disease, even when using different time series. Our estimates are consistent with previous studies based on secondary case data. Quantifying the contribution of super-spreading to disease transmission has important implications for infectious disease management and control. Our modelling framework is disease-agnostic and implemented as an R package, with potential to be a valuable tool for public health.

## 1 Introduction

When an epidemic outbreak occurs, the transmission dynamics are rarely homogeneous. During the Covid-19 pandemic of SARS-CoV-2 for example, it became evident that super-spreading events played a crucial role in the outbreak and early on signs of super-spreading were reported (Du et al., 2022; Endo, Abbott, Kucharski, & Funk, 2020; Wang et al., 2020). Events such as weddings, family gatherings and sports events, in which many people are infected at once, spawned dangerous outbreaks (Lewis, 2021). Such uneven transmission dynamics are common among the Coronavirus's relatives, including SARS-CoV-1, responsible for the severe acute respiratory syndrome (SARS) epidemic in 2003, and MERS-CoV, which causes the Middle East respiratory syndrome (Brainard et al., 2023; Wang et al., 2021).

A key parameter in understanding the transmission dynamics is the basic reproduction number *R*_0_, the average number of secondary infections caused by one infected individual in a population where all individuals are susceptible to infection. An estimate of *R*_0_ can help establish if there is a high probability of a major outbreak occurring and can provide feedback on the success of control interventions given that the goal of such efforts is to reduce *R*_0_ below the threshold value of 1 (Ferguson et al., 2006; Fraser et al., 2004, Fraser et al., 2009). The most common modelling approach in epidemics is to use a transmission or compartmental model (Keeling & Rohani, 2011). However, branching process models are a flexible and biologically-realistic alternative (Farrington et al., 2003). They are particularly suited for modelling the early stages of an outbreak, for studying *R*_0_ and transmission heterogeneity which is why we used them in this research. For example the widely used epidemic modelling tool developed by Cori et al. (2013) uses a branching process model to estimate *R*_*t*_, the time-varying reproduction number over the course of an epidemic. The framework is disease agnostic and uses as input case incidence data, as we do here, as opposed to contact tracing or secondary case data, which is less readily available.

The modelling of super-spreading in epidemics is present in the literature but remains relatively limited (Grassly & Fraser, 2008). The work of Lloyd-Smith et al. (2005) was a seminal paper on this topic. The authors compared the fit of three different offspring distributions – Poisson, geometric and negative binomial – to data on the number of secondary cases. However this approach requires access to contact tracing data, which is not often readily available. Furthermore the negative binomial distribution, despite being the best fitting model, fails to capture possible bimodality of the offspring distribution. More recently, the work of Ho et al. (2023) on super-spreading and over-dispersion also applied the negative binomial, but directly to incidence data to estimate *R*_*t*_. However, no comparison is made with competing alternate models. Here we perform Bayesian inference and comparison between five models: a baseline model without any transmission heterogeneity, two models of super-spreading individuals (unimodal and bimodal) and two models of super-spreading events (unimodal and bimodal). We apply this framework to several simulated and real datasets to showcase its usefulness. This paper directly extends the first author's doctoral thesis (Craddock, 2024). Given the documented prominence of super-spreading in the recent epidemics of the 21st century, quantifying the contribution of super-spreading on disease transmission has important implications for the control and management of infectious diseases.

## 2 Material and methods

### 2.1 Modelling framework overview

We present a framework of five discrete-time, stochastic branching process models to describe infectious disease transmission through a susceptible population. The epidemiological process considered is a branching process whereby each infected individual transmits to secondary cases called offspring (Farrington et al., 2003). The framework is adaptable to a range of infectious diseases. Our models are fit to incidence data, which represents reported cases over time (e.g., daily cases). We define the incidence data *I*_*t*_ as the number of infections that occur at time *t* and the total incidence data up to time *T* as ***I***_[1:*T*]_ = [*I*_1_, *I*_2_, *…*, *I*_*T*_]. The time *t* is measured in arbitrary units of calendar time, for example days or weeks.

**Fig. 1:**
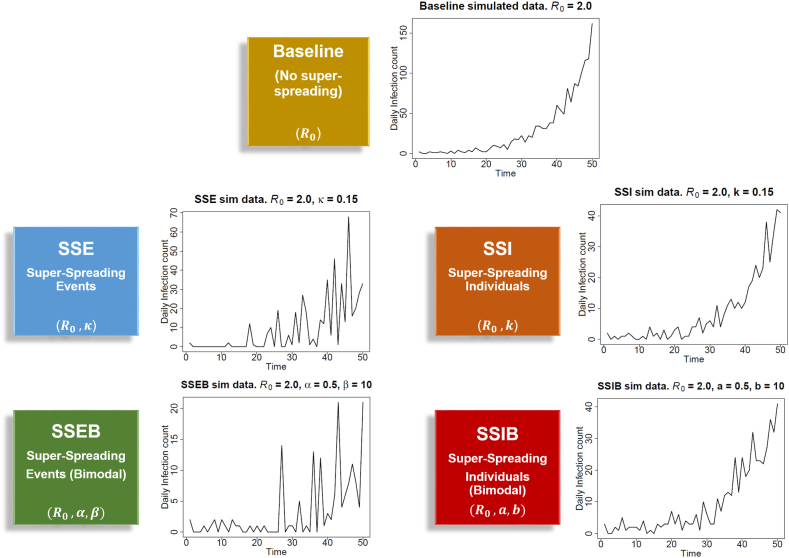
Typical simulation from the five models of epidemic transmission. The time series plots show incidence data ***I***_[1:*T*]_ simulated from the Baseline, SSE, SSI, SSEB and SSIB models for *R*_0_ = 2.0, duration *T* = 50.

To account for super-spreading or over-dispersion in transmission, the negative binomial model is often chosen to model the offspring distribution *Z*, describing the number of secondary infections per individual (Endo, Abbott, Kucharski, & Funk, 2020; Lloyd-Smith et al., 2005). However, this model is uni-modal and so it is unable to generate multiple or even secondary modes to account for additional super-spreading phenomena. We seek to address this limitation by introducing two novel bimodal models of epidemic transmission. Our model framework of epidemic transmission consists of a Baseline model with no super-spreading properties, two models that describe super-spreading events (SSE and SSEB) and two models that describe super-spreading individuals or infections (SSI and SSIB). We define a super-spreading event (SSE) as an event or point in time in which a large number of infections are generated. Such events could include weddings, family gatherings and sports events, in which many people were infected at once (Lewis, 2021). A super-spreading individual (SSI) refers to an individual who is more infectious than non super-spreading individuals in the population. Wallinga and Teunis (2004) defines such a super-spreading individual as one that produces at least 10 secondary infections. The models also vary by mode; the Baseline, SSE, and SSI models are uni-modal, using the Poisson and negative binomial distributions. The bimodal (suffix ‘B’) models, the SSEB and SSIB models, introduced in this work, offer novel approaches for modelling super-spreading events and individuals. Typical simulations from each of the five models are displayed in Fig. 1. For the super-spreading events models (SSE and SSEB), the spikes in infections at certain time-points corresponding to super-spreading events are evident. For the SSI and SSIB models, the increased infectivity of super-spreading individuals lasts the duration of their infectious period, so spikes in infections do not occur at a specific time point, but rather are spread across the duration of their infectivity. The models are detailed below in turn and summarized in Table 1. Generating incidence data *I*_*t*_ at time *t* from each model requires considering the infectivity of all individuals infected at earlier time points. We assume that each infected individual has an infectivity profile given by a probability distribution denoted *ω*(*τ*), dependent on the time since infection time *τ*, as in many previous studies (Wallinga & Teunis, 2004; Cori et al., 2013; Didelot et al., 2017). The distribution *ω*(*τ*) is also known as the generation time distribution, and represents the time interval between infection of a primary case and one of its secondary cases (ie directly infected by the primary case). For example if a primary case causes three secondary infections (*Z* = 3), then the marginal distributions of the infections times follow the generation time distribution. However, in the SSE model the infection times are clustered together, whereas in the SSI (and baseline) models the infection times would be independent and identically distributed draws from the generation time distribution.

**Table 1:** The five epidemic transmission models fit to incidence data, their parameters and distributions used. For *R*_0_ and *k* the ‘parameter range of interest’ encompasses the range of estimates from various modeling studies of different diseases as summarized in Table I of the Supplementary Materials.

Model	Parameters	Parameter Range of Interest	Incidence Data
**Baseline** No Super-spreading	*R*_0_: Basic Reproduction Number	(0, 10]	*I*_*t*_ ∼ Poisson(*R*_0_*λ*_*t*_)
**SSE** Super-spreading events	*R*_0_*k*: DispersionParameter	(0, 10] (0, 1]	*I*_*t*_ ∼ NegBin(*r* = *kλ*_*t*_, *μ* = *R*_0_*λ*_*t*_)
**SSI** Super-spreading individuals	*R*_0_*k*	(0, 10] (0, 1]	$I_{t}|{\nu^{+}}_{\left[1:t-1\right]}∼\text{Poisson}\left(\sum_{\tau =1}^{t-1}\nu_{\tau }^{+}\cdot \omega \left(t-\tau \right)\right)$${\nu^{+}}_{\left[1:T\right]}|I_{t}∼Gamma\left(I_{t}\cdot k,R_{0}/k\right)$${\nu^{+}}_{t}$: sum of individual reproduction numbers *ν* on day *t*
**SSEB** Super-spreading events bimodal	*R*_0_*α*: Proportion of *R*_0_ due to non-SSE infections *β*: Average size of a SSE	(0, 10] [0, 1] (1, 20]	*I*_*t*_ = *N*_*t*_ + *S*_*t*_*N*_*t*_ ∼ Poisson(*α* ⋅ *R*_0_ ⋅ *λ*_*t*_) Infections from Non SSEs *S*_*t*_ ∼ Poisson(*β* ⋅ *E*_*t*_) Infections from SSEs *E*_*t*_ ∼ Poisson(*R*_0_(1 − *α*)/*β* ⋅ *λ*_*t*_) Number of SSEs
**SSIB** Super-spreading individuals bimodal	*R*_0_*a*: Proportion of *R*_0_ due to non-SSI infections *b*: Increased infectivity of SSIs	(0, 10] [0, 1] (1, 20]	*I*_*t*_ = *N*_*t*_ + *S*_*t*_$N_{t}∼\text{Poisson}\left(aR_{0}\lambda_{t}^{\prime }\right)$ Non-SSI Infections $S_{t}∼\text{Poisson}\left(R_{0}\left(1-a\right)/b\cdot \lambda_{t}^{\prime }\right)$ SSI Infections $\lambda_{t}^{\prime }=\sum_{\tau =1}^{t-1}\left(N_{\tau }+b\cdot S_{\tau }\right)\cdot \;\omega \left(t-\tau \right)$

Let *λ*_*t*_ denote the sum of the contributions of the generation time distributions for all individuals infected at earlier time points to infections at time *t*. Each individual infected at time *τ* ∈ [1, …, *t* − 1] contributes *ω*(*t* − *τ*) to *λ*_*t*_. We assume no infectivity from individuals infected prior to time *t* = 1. The value of *λ*_*t*_ is therefore defined as:

(1) $$\lambda_{t}=\overset{t-1}{\underset{\tau =1}{\sum }}I_{\tau }\cdot \omega \left(t-\tau \right)$$

### 2.2 The baseline model

The Poisson model is commonly used to capture the stochasticity of epidemic transmission (Diekmann & Heesterbeek, 2000; Lloyd-Smith et al., 2005), however as its mean equals its variance, it cannot capture transmission heterogeneity. Thus, it serves as our baseline for comparison. The offspring distribution of an individual is:

(2) $$Z∼\text{Poisson}\left(R_{0}\right)$$

The mean of a Poisson distribution is equal to the parameter *R*_0_ which is the only parameter of this model. To generate incidence data *I*_*t*_ from the Baseline model, we use the fact that a sum of Poisson-distributed random variables is also Poisson-distributed, so that:

(3) $$I_{t}∼\text{Poisson}\left(R_{0}\cdot \lambda_{t}\right)$$

Full details of the Baseline model derivation are provided in Section 2.1 of the Supplementary Materials.

### 2.3 The SSE model

The SSE model is a unimodal model for super-spreading events. Unlike the Poisson distribution, the negative binomial distribution has differing mean and variance, allowing for over-dispersion in the number of secondary infections transmitted. A key parameter of this model is the dispersion parameter *k*, a parameter used to quantify heterogeneity in certain distributions (Lloyd-Smith, 2007). In the SSE model the offspring distribution is defined as:

(4) $$Z∼\text{NegativeBinomial}\left(r=k,\mu =R_{0}\right)$$

While the negative binomial distribution is commonly applied to *Z*, its use for *I*_*t*_ is less frequent. A notable example is Ho et al. (2023), where incidence data is employed to estimate *R*_*t*_, the time-varying reproduction number. In our SSE model, we adopt a similar parameterization involving *R*_0_, parameterized by size *r* and mean *μ*, with parameters *R*_0_ and *k*:

(5) $$I_{t}∼\text{NegativeBinomial}\left(r=k\lambda_{t},\mu =R_{0}\lambda_{t}\right)$$

We provide full details of the SSE model derivation in Section 2.2 of the Supplementary Materials. In this parameterization we note that as the dispersion parameter *k* decreases, the variance increases, signaling greater transmission heterogeneity and potential for super-spreading (Didelot et al., 2025; Du et al., 2020). Accurate estimation of *k* is crucial for determining the need for public health measures; significant outbreaks may occur when *k* is small, even if *R*_0_ < 1 (Kucharski & Althaus, 2015).

### 2.4 The SSI model

The SSI model is a unimodal model for super-spreading individuals. In this model super-spreading individuals arise from the right-hand tail of the infectivity distribution rather than forming a distinct group. Building on Lloyd-Smith et al. (2005), this model uses the same negative binomial distribution for *Z* and extends it by introducing incidence data *I*_*t*_ derived from the model. In Lloyd-Smith et al. (2005), the infectivity *ν* is introduced as a random variable representing the expected number of secondary cases from an individual, with *Z* ∼ Poisson(*ν*). Unlike simpler models where *ν* = *R*_0_, the SSI model assumes *ν* is drawn from a Gamma distribution with mean *R*_0_ and shape and scale parameters *α* and *θ*, respectively:

(6) $$\nu ∼Gamma\left(\alpha =k,\theta =\frac{R_{0}}{k}\right)$$

The offspring distribution *Z* follows a Poisson distribution with rate *ν* which is gamma distributed and is therefore a negative binomial random variable:

(7) $$Z∼\text{NegativeBinomial}\left(r=k,\mu =R_{0}\right)$$

As before, small values of *k* correspond to high levels of heterogeneity in transmission. The offspring distribution *Z* is the same as in the SSE model, however the models differ in how the incidence data *I*_*t*_ is derived. To generate incidence data *I*_*t*_ from the SSI model, we introduce a new variable $\nu_{t}^{+}$ as the sum of all individual reproduction numbers *ν*_*i*_ of each individual *i* infected at time *t*: $\nu_{t}^{+}=\sum_{i=1}^{\infty }\nu_{i}1_{\left\{i\text{ infected on day t}\right\}}$. By applying the scaling properties of the gamma distribution to the distribution of *ν* in Equation (6) and accounting for all infected individuals on day *t*, we derive:

(8) $$\nu_{t}^{+}|I_{t}∼Gamma\left(\alpha =I_{t}\cdot k,\theta =R_{0}/k\right)$$

We record $\nu_{t}^{+}$ for the duration of the epidemic in the following vector ${\nu^{+}}_{\left[1:T\right]}=\left[\nu_{1}^{+},\nu_{2}^{+},\ldots ,\nu_{t}^{+},\ldots ,\nu_{T}^{+}\right]$. Incidence data *I*_*t*_ from the SSI model depends on past $\nu_{t}^{+}$ values. The incidence data *I*_*t*_ follows a Poisson distribution with a rate based on the gamma-distributed $\nu_{t}^{+}$:

(9) $$I_{t}|{\nu^{+}}_{\left[1:t-1\right]}∼Poisson\left(\overset{t-1}{\underset{\tau =1}{\sum }}\nu_{\tau }^{+}\cdot \omega \left(t-\tau \right)\right)$$

Once *I*_*t*_ is generated at time *t*, we draw $\nu_{t}^{+}$ using Equation (8). The generation of incidence data from the SSI model, assuming known incidence data *I*_1_ at time *t* = 1 and model parameters *R*_0_ and *k*, is achieved by iteratively sampling from the last two equations.

### 2.5 The SSEB model

The SSEB model is a bimodal model for super-spreading events. In the SSEB model, the total infections at time *t*, *I*_*t*_, arise from two mechanisms: homogeneous transmission and super-spreading. These are represented as two independent discrete-time processes, *N*_*t*_ (homogeneous) and *S*_*t*_ (super-spreading), such that *I*_*t*_ = *N*_*t*_ + *S*_*t*_. The number of super-spreading events at time *t* is denoted *E*_*t*_, each of which causes an increased number of infections captured by the parameter *β*. The parameter *α* is the proportion of *R*_0_ attributable to homogeneous transmission, with 1 − *α* representing the contribution from super-spreading events. *β* reflects the increased number of infections for each super-spreading event.

The incidence data from non-SSE events follows a Poisson distribution:

(10) $$N_{t}∼\text{Poisson}\left(\alpha \cdot R_{0}\cdot \lambda_{t}\right)$$

The number of infections resulting from super-spreading events at time *t* is denoted *S*_*t*_. As previously mentioned, *E*_*t*_ denotes the total number of such super-spreading events at time *t*, for example a concert or a wedding (Adam et al., 2020). Each event contributes an increased number of infections, quantified by the parameter *β*. Specifically, each super-spreading event results in a Poisson-distributed number of infections with mean *β*. It follows that *S*_*t*_ is a compound Poisson distribution (Adelson, 1966). The contribution to *R*_0_ from super-spreading events is *R*_0_(1 − *α*), and since an SSE yields *β* times more infections:

(11) $$E_{t}∼Poisson\left(\frac{R_{0}\left(1-\alpha \right)}{\beta }\cdot \lambda_{t}\right)$$

(12) $$S_{t}|\left(E_{t}=e_{t}\right)∼Poisson\left(\beta \cdot e_{t}\right)$$

For the purposes of implementation let the maximum possible number of super-spreading events at any given time *t* be *M* events, we can then decompose:

(13) $$p\left(S_{t}=s_{t}\right)=\overset{M}{\underset{e_{t}=0}{\sum }}p\left(E_{t}=e_{t}\right)\cdot p\left(S_{t}=s_{t}|E_{t}=e_{t}\right)$$

The total number of infections *I*_*t*_ at each point in time is *I*_*t*_ = *N*_*t*_ + *S*_*t*_ and by the addition of two Poisson distributions that are assumed to be independent, i.e Equation (10) and Equation (12) the incidence data of the SSEB model is defined to be:

(14) $$I_{t}|\left(E_{t}=e_{t}\right)∼\text{Poisson}\left(\alpha R_{0}\lambda_{t}+\beta e_{t}\right)$$

In the SSEB model, the offspring distribution of a single individual encompasses the contribution due to non super-spreading events and the contribution due to super-spreading events as follows:

(15) $$Z∼\text{Poisson}\left(\alpha R_{0}\right)+\text{Poisson}\left(\beta \left(\text{Poisson}\left(\frac{R_{0}\left(1-\alpha \right)}{\beta }\right)\right)\right)$$

The second term is a compound Poisson distribution. We can check that the expectation of *Z* is $E\left[Z\right]=\alpha R_{0}+\beta \cdot \frac{R_{0}\left(1-\alpha \right)}{\beta }=\alpha R_{0}+R_{0}\left(1-\alpha \right)=R_{0}$.

### 2.6 The SSIB model

The SSIB model is a bimodal model for super-spreading individuals. The model features two classes of infected individuals: non-super-spreading individuals *N* and super-spreading individuals *S*, the latter being *b* times more infectious. Both classes can produce offspring that are either super-spreading individuals or not. The incidence data *I*_*t*_ at time *t* comprises non super-spreading individuals (infections), *N*_*t*_ and super-spreading individuals (infections) *S*_*t*_. The parameter *a* represents the proportion of *R*_0_ attributed to non-super-spreading individuals, analogous to *α* in the SSEB model. The parameter *b* denotes the increased infectiousness of super-spreading individuals. Their infectivity profile is increased by a factor *b*, which is incorporated into the updated $\lambda_{t}^{\prime }$. The parameter $\lambda_{t}^{\prime }$ depends on the history of non super-spreading infections and super-spreading infections as follows:

(16) $$\lambda_{t}^{\prime }|\left(N_{\left[1:t-1\right]},S_{\left[1:t-1\right]}\right)=\overset{t-1}{\underset{\tau =1}{\sum }}\left(N_{\tau }+b\cdot S_{\tau }\right)\cdot \omega \left(t-\tau \right)$$

The incidence data *I*_*t*_ = *N*_*t*_ + *S*_*t*_ comprises *N*_*t*_ non super-spreading individuals and *S*_*t*_ super-spreading individuals distributed as:

(17) $$N_{t}∼\text{Poisson}\left(a\cdot R_{0}\cdot \lambda_{t}^{\prime }\right)$$

(18) $$S_{t}∼\text{Poisson}\left(\frac{R_{0}\left(1-a\right)}{b}\cdot \lambda_{t}^{\prime }\right)$$

To compute *Z* we need to account for the contribution of transmission from both *N* and *S*. For a non-super-spreading individual *N*, their offspring can be either non super-spreading individuals, contributing *Z*_*N*|*N*_ to *Z*, or super-spreading individuals, contributing *Z*_*S*|*N*_ to *Z*:

(19) $$Z_{N|N}+Z_{S|N}∼\text{Poisson}\left(aR_{0}\right)+\text{Poisson}\left(\frac{\left(1-a\right)R_{0}}{b}\right)=\text{Poisson}\left(aR_{0}+\frac{\left(1-a\right)R_{0}}{b}\right)$$

For a super-spreading individual *S*, their offspring can either be non super-spreading individuals, contributing *Z*_*N*|*S*_ to *Z*, or super-spreading individuals, contributing *Z*_*S*|*S*_ to *Z*:

(20) $$Z_{N|S}+Z_{S|S}∼\text{Poisson}\left(abR_{0}\right)+\text{Poisson}\left(\left(1-a\right)R_{0}\right)=\text{Poisson}\left(abR_{0}+\left(1-a\right)R_{0}\right)$$

The probability *p* of being a super-spreading individual is $p=\frac{\frac{1-a}{b}R_{0}}{\frac{1-a}{b}R_{0}+aR_{0}}=\frac{1-a}{1-a+ab}$. The total offspring distribution *Z* is a mixture of the two distributions with weights 1 − *p* and *p* respectively:

(21) $$Z∼\left(1-p\right)\text{Poisson}\left(aR_{0}+\frac{\left(1-a\right)R_{0}}{b}\right)⨁p\text{Poisson}\left(abR_{0}+\left(1-a\right)R_{0}\right)$$

We can check that the expectation of *Z* is $E\left[Z\right]=\left(1-p\right)\left(aR_{0}+\frac{\left(1-a\right)R_{0}}{b}\right)+p\left(abR_{0}+\left(1-a\right)R_{0}\right)=R_{0}$.

### 2.7 Bayesian inference methodology

We use a Bayesian framework for inference of model parameters. We sample from the posterior distributions of the model parameters using Markov Chain Monte-Carlo (MCMC) methods. Consider a model *M* with parameters ***θ*** = (*θ*_1_, *θ*_2_, …, *θ*_*p*_) and incidence data ***I***_[1:*T*]_ = [*I*_1_, *I*_2_, *…*, *I*_*T*_]. In Bayesian inference, prior information *p*(***θ***|*M*) about the parameters ***θ*** is combined with information from the sample data contained within the likelihood *p*(***I***_[1:*T*]_|***θ***, *M*). The result of updating the prior distribution based on observed data is called the posterior distribution *p*(***θ***|***I***_[1:*T*]_, *M*). The posterior distribution contains all the available information about ***θ***, is used to make estimates or inferences and allows us to quantify the uncertainty associated with our parameter estimates. In selecting our priors, we primarily opt for weakly informative priors to guide our analysis. These priors allow a balance between existing knowledge and the data-driven inference process. Our framework remains disease-agnostic, allowing for the selection of more informative priors tailored to specific datasets as required. The prior distributions chosen for our models are displayed in Table 2. For the parameter *R*_0_, common to all five models, an Exponential(1) distribution is used as the prior distribution throughout the study. This distribution with a mean centered on 1 implies that a priori, we are not specifying whether the outbreak is likely to propagate throughout the population, *R*_0_ > 1, or die out, *R*_0_ < 1. A comprehensive explanation behind our choice of prior distributions is available in Section 3 of the Supplementary Materials and the results of our prior sensitivity analysis are presented in Section 4. The MCMC algorithms used for inference of the five models are all variations of the Metropolis Hastings algorithm. Data augmentation is required to evaluate the likelihoods of the SSI and SSIB models which is necessary to carry out inference of the models. The algorithms in full are detailed in (Craddock, 2024).

**Table 2:** The model parameters, their chosen prior distributions and the relevant metrics of the chosen distributions including the support and mean.

Models	Parameter	Prior Distribution	Prior support	Prior Mean
All	*R*_0_	Exponential(1)	[0, *∞*]	1
SSE, SSI	*k*	Exponential(5)	[0, *∞*]	0.2
SSEB, SSIB	*a*, *α*	Beta(2,2)	[0, 1]	0.5
SSEB, SSIB	*b*, *β*	1 + Gamma(3,3)	[1, *∞*]	10

### 2.8 Model comparison

Model comparison is the process of comparing a set of candidate models, given data (Linhart & Zucchini, 1986). In Bayesian statistics this is achieved by computing Bayes factors which is a ratio of model evidence (Kass & Raftery, 1995; Hoeting et al., 1999). Given model *M*, data ***I***_[1:*T*]_, parameter vector ***θ***, parameter prior *p*(***θ***|*M*) and likelihood *p*(***I***_[1:*T*]_|***θ***, *M*), the model evidence is *p*(***I***_[1:*T*]_|*M*) = *∫p*(***I***_[1:*T*]_|***θ***, *M*)*p*(***θ***|*M*) *d****θ***. Posterior model probabilities, *p*(*M*_*i*_|***I***_[1:*T*]_), provide a comprehensive comparison of multiple models by normalizing their Bayes factors (Ando, 2010) as defined in Equation (22). In this equation *p*(*M*_*i*_) represents the prior probability on model *M*_*i*_. If no prior preference exists, the model prior is often uniform over all *m* models; *p*(*M*_*j*_) = 1/*m*. Using this prior, selecting the most likely model reduces to choosing the one with the highest evidence.

(22) $$p\left(M_{i}|I_{\left[1:T\right]}\right)=\frac{p\left(I_{\left[1:T\right]}|M_{i}\right)p\left(M_{i}\right)}{\sum_{j=1}^{m}p\left(I_{\left[1:T\right]}|M_{j}\right)p\left(M_{j}\right)}$$

To estimate the model evidence, we use a method that combines importance sampling with MCMC, as proposed by Touloupou et al. (2018). This approach has been shown to be effective, especially in scenarios with missing data (Hudson et al., 2023; McKinley et al., 2020). Importance Sampling (IS) can be used to estimate properties of a target distribution by generating weighted samples from a proposal distribution that is generally easier to sample from. Given data ***x*** and proposal distribution *q* the marginal likelihood can be written as:

(23) $$p\left(x\right)=\int_{\Theta }p\left(x|\theta \right)\frac{p\left(\theta \right)}{q\left(\theta \right)}q\left(\theta \right)\;d\theta$$

An unbiased estimator of the model evidence is therefore:

(24) $$\hat{p}\left(x\right)=\frac{1}{N}\overset{N}{\underset{i=1}{\sum }}p\left(x|\theta_{i}\right)\frac{p\left(\theta_{i}\right)}{q\left(\theta_{i}\right)}$$

The effectiveness of the estimator depends upon the variance of the sampling estimate as outlined in Touloupou et al. (2018). To minimize the variance, we want the proposal *q*(***θ***) to resemble the posterior as closely as possible. The proposal distribution *q*(***θ***) is typically derived from the MCMC output. Clyde et al. (2007) suggests using a multi-variate *t*-distribution with the location and scale parameters estimated from the MCMC output. To ensure the proposal is over-dispersed relative to the target distribution we use a “defense mixture”, which reduces variance (Hesterberg, 1995). The mixing proportion *p* in the defense mixture is typically set to 0.95, ensuring the ratio of prior to proposal density remains bounded above by 1/(1 − *p*) Hesterberg (1995). For priors, we use weakly informative Exponential(1) priors for model parameters, similar to Hudson et al. (2023). The defense mixture is:

(25) $$q_{\text{D}}\left(\theta \right)=p\cdot q\left(\theta \right)+\left(1-p\right)\cdot p\left(\theta \right)$$

To obtain an estimate of the model evidence, $\hat{p}\left(I_{\left[1:T\right]}\right)$ for each of the five models we use the following steps based on Touloupou et al. (2018). (1) Obtain samples ***θ*** from the posterior distribution *p*(***θ***|***I***_[1:*T*]_, *M*_*i*_) by fitting model *M*_*i*_ to incidence data ***I***_[1:*T*]_ using MCMC. (2) Derive the proposal distribution *q*(⋅), a parametric approximation of the posterior distribution, using the sample ***θ***. For the uni-variate Baseline model, a student's t-distribution with three degrees of freedom is used. For the four multivariate models (SSE, SSI, SSEB, SSIB) a multivariate t-distribution (with three degrees of freedom) is used as the proposal distribution. For the SSIB model the proposal distribution is more involved. This is due to the augmented data; the super-spreading infections data ***S***_[1:*T*]_ required for evaluation of the model's likelihood. For the model's continuous parameters (*R*_0_, *a*, *b*) the multivariate t-distribution is used as above. For ***S***_[1:*T*]_, a Dirichlet multinomial distribution is chosen as the proposal distribution. This distribution is often used to model categorical data. (3) Estimate the model evidence $\hat{p}\left(I_{\left[1:T\right]}\right)$ as in Equation 24. (4) Repeat steps (1)–(3) to estimate the model evidence for all five models in the framework; $\hat{p}\left(I_{\left[1:T\right]}|M_{\text{Baseline}}\right),\hat{p}\left(I_{\left[1:T\right]}|M_{\text{SSE}}\right),\hat{p}\left(I_{\left[1:T\right]}|M_{\text{SSI}}\right),\hat{p}\left(I_{\left[1:T\right]}|M_{\text{SSEB}}\right)\text{ and }\hat{p}\left(I_{\left[1:T\right]}|M_{\text{SSIB}}\right)$. (5) To carry out model comparison, the posterior model probabilities are calculated for all five models using Equation (22). The model with the highest posterior probability is selected as the best-fitting model.

### 2.9 Implementation

We implemented the analytical methods described in this paper in a new R package which is available at https://github.com/hanmacrad2/SuperSpreadingEpidemicsMCMC for R version 3.5 or later. All code and data needed to replicate the results are included in this repository.

## 3 Results

### 3.1 Parameter inference on simulated data

**Fig. 2:**
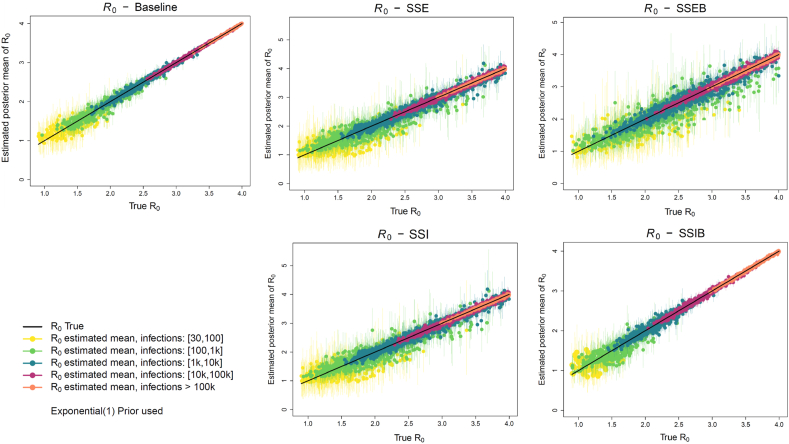
Results of inference of *R*_0_ across the five models. Posterior estimates in each model fit to 5000 simulated datasets from the model itself for *R*_0_ in the range [0.9, 4.0]. Dots indicate the mean of the estimated posteriors, bars represent the 95 % CI of the posterior, and the black diagonal line represents the true value used for simulation.

An extensive simulation study was carried out to assess inference performance. Simulation studies provide the unique advantage of knowing the underlying values used for generating the data, which can be used for comparison and validation of the estimates (Geweke, 2004). The results of one such simulation study are displayed in Fig. 2. The accuracy and precision of inference of the five models (Baseline, SSE, SSI, SSEB, SSIB) in inferring *R*_0_ across a range of relevant values are assessed allowing for a robust evaluation of inference performance across varying epidemic scenarios and data conditions. The results are based on 5000 simulated datasets generated from the models themselves with *R*_0_ simulated across the range [0.9 − 4.0]. This range for *R*_0_ encompasses the estimates presented in Table Iof the Supplementary Materials, which synthesizes findings from a range of modeling studies.

In general inference is working well across the five models. The total infection count of each simulated epidemic has an effect on the inferred results, as indicated by the color-coded simulations. Higher infection counts (magenta, orange) have smaller biases and tighter 95 % CI compared to lower counts (yellow, green).

Table 3 contains the bias and coverage metrics for the inference of each parameter of each model. The parameters used in the simulations were drawn uniformly from the range indicated. These results represent the mean values across 5000 independent repetitions of MCMC inference applied to 5000 different simulations from each model. The simulations are of duration 50 days. The bias (i.e. the mean difference between the parameter used in simulation and posterior mean) is small, in particular for *R*_0_. It is slightly higher for the *β* parameter of SSEB and the *b* parameter of SSIB, mostly because the natural range of these parameters is wider. The coverage (i.e. probability that the 95 % CI contains the correct value used in the simulation) is around 95 % for all parameters of all models, as expected under ideal conditions when the simulation and inference models are the same. A prior sensitivity analysis of Bayesian parameter inference is presented in Section 4 of the Supplementary Materials.

**Table 3:** Performance metrics from the inference of parameters across the five epidemic models.

Model	Parameter	Range Tested	Bias	95 % Coverage
Baseline	*R*_0_	[0.9, 4.0]	−0.007	94
SSE	*R*_0_	[0.9, 4.0]	−0.017	94
	*k*	[0.01, 0.2]	0.01	96
SSI	*R*_0_	[0.9, 4.0]	−0.016	94
	*k*	[0.05, 0.2]	0.09	84
SSEB	*R*_0_	[0.9, 4.0]	−0.02	95
	*α*	[0, 1]	0.0003	95
	*β*	[5, 15]	−0.16	97
SSIB	*R*_0_	[0.9, 4.0]	−0.009	93
	*a*	[0, 1]	−0.05	92
	*b*	[5, 15]	0.35	96

### 3.2 Model comparison on simulated data

**Fig. 3:**
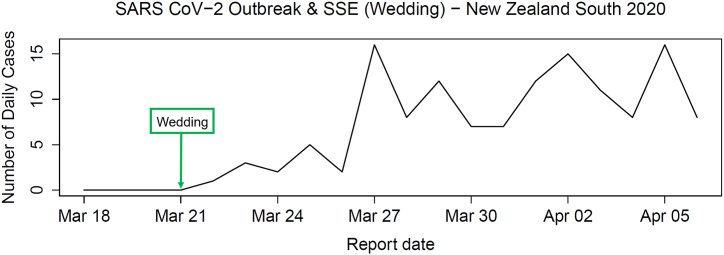
Incidence data from New Zealand's Southland district during the SARS-CoV-2 outbreak in 2020, before and after the wedding-related super-spreading event on March 21st.

**Fig. 4:**
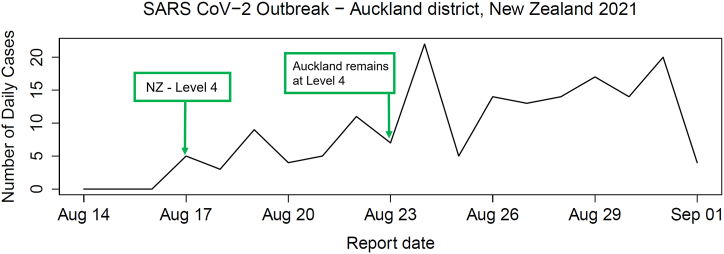
Reported cases from the Waitemata district of Auckland, New Zealand during a wave of SARS-CoV-2 cases in August 2021 resulting in a Level 4 lockdown.

**Fig. 5:**
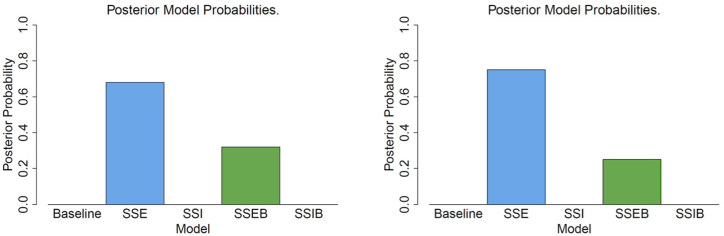
Bar plots of the posterior model probabilities of the five models applied to the two waves of SARS-CoV-2, New Zealand in 2020 and 2021.

**Fig. 6:**
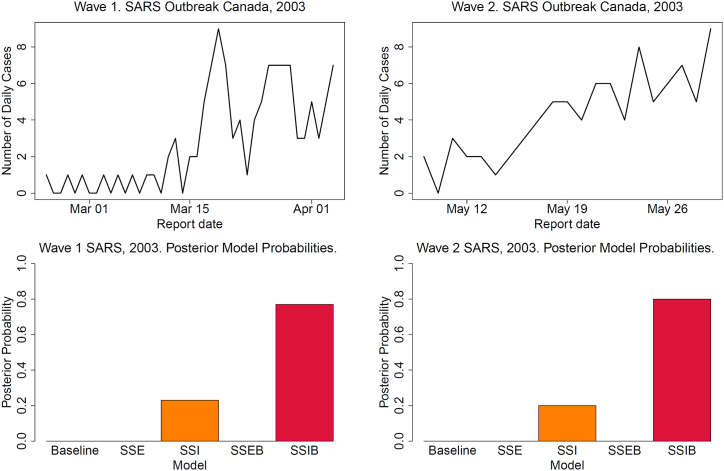
Incidence data and bar plots of the posterior model probabilities of the five models applied to the two waves of SARS outbreaks in 2003.

To test our model comparison methodology we perform a simulation study using data generated from the five models before considering applications to real data. The goal is to determine how consistently model selection can select the correct simulation model as the most likely candidate among the five. For each analysis, we select one model as the simulation model, simulate 100 incidence datasets, and fit all five models to compute posterior model probabilities using Equation (22). For the prior model weight we put equal probability a priori on each model and so *p*(*M*_*i*_) = 0.2. The results of a simulation study are summarized in Table 4. The table contains posterior model probabilities (mean, CI) of the simulation study for all combinations of simulation model and fitted model. Consistently, the model with the highest posterior probability is the simulation model, aligning with our goal. For *n* = 100 simulations from the Baseline model, the Baseline model was selected 99 % of the time, with a mean posterior probability of 0.94 (CI: [0.77, 0.99]). Similarly, the SSE model was identified 93 % of the time (mean: 0.90, CI: [0.43, 1.0]), the SSI model 67 % of the time (mean: 0.63, CI: [0.06, 1.0]), the SSEB model 97 % of the time (mean: 0.896, CI: [0.68, 1.0]), and the SSIB model 63 % of the time (mean: 0.554, CI: [0.05, 0.99]). A complete visual representation of the posterior model probabilities is available in the Supplementary Materials (Fig. 1, Fig. 2, Fig. 3, Fig. 4, Fig. 5, Fig. 6).

**Table 4:** Summary of estimated posterior model probabilities (mean and CI) for each combination of simulated model and fitted model for *N* = 100 simulations from each model in the left hand column. These results are visualised in full in the Supplementary Materials (Figs. I-IV).

Simulation Model	Selection Probability	Baseline	SSE	SSI	SSEB	SSIB
Baseline	99 %	**0.94 [0.77, 0.99]**	0.001	0.024	0.034	0.001
SSE	93 %	0	**0.90 [0.43, 1.0]**	0	0.10	0
SSI	67 %	0.09	0.02	**0.63 [0.06, 1.0]**	0.10	0.152
SSEB	97 %	0.002	0.101	0	**0.896 [0.68, 1.0]**	0.001
SSIB	63 %	0.19	0	0.232	0.023	**0.555 [0.05, 0.99]**

These results indicate strong performance in identifying the correct model, especially for the Baseline, SSE, and SSEB models, with some variability observed for the SSI and SSIB models, reflecting greater uncertainty. An additional result is that the super-spreading events models identify each other as the second most probable models, as do the super-spreading individuals models. These findings support our strategy of developing separate models of super-spreading events and super-spreading individuals and treating the two mechanisms as separate modes of super-spreading in relation to epidemic outbreaks. We also perform a prior sensitivity analysis, repeating the model comparison with both the original priors and uniform priors, across various scenarios, including different *R*_0_ values and epidemic durations (see Craddock (2024)).

### 3.3 SARS-CoV-2 outbreaks, New Zealand, 2020–2021

We now turn to the application of our modelling framework to real epidemic outbreaks. We fit our models to the reported incidence datasets and infer the model parameters using our MCMC algorithms. We then apply our model comparison framework to determine the most likely model or ‘maximum a posteriori’ of our five models when fit to the reported incidence data. Cases from the first time step are assumed to be imported cases, as in comparable methods (Cori et al., 2013).

We first focus on outbreaks of SARS-CoV-2 in Aotearoa New Zealand in 2020 and 2021. In early 2020, New Zealand implemented a strict nationwide lockdown and closed its borders to all non-New Zealanders on March 20th^,^ 2020 (Cumming, 2022) aiming for virus elimination (Geoghegan et al., 2020). As a geographically isolated island with borders that can be easily sealed, once closed, transmission could only occur at local or national level rather than from imported cases. The country also maintained a vigilant record of incidence cases throughout the pandemic and followed a four-level Alert Level framework (Cumming, 2022; Department of the Prime Minister and Cabinet, 2024). For these reasons we chose incidence data from New Zealand as one of the focuses of our analysis. For SARS-CoV-2 Li et al. (2020) estimated a mean serial interval of 7.5 days, 95 % CI (5.3, 19 days). Nishiura et al. (2020) examined early cases of Covid-19 in China and estimated the mean serial interval to be 4.7 days 95 % CI (4.5, 4.9 days). We use a Gamma(6,1) distribution, with a mean of 6 and mode of 5, as it fits within the credible intervals of the serial interval estimates.

In March 2020, a wedding in Bluff, Southland, led to New Zealand's largest cluster at the time, with 87 infections, and was classified as a super-spreading event (New Zealand Herald, 2020; Stuff, 2020). We use case data from Southland before and after the event (Fig. 3) (Ministry of Health New Zealand, 2024). No cases were reported before March 21st, followed by a sharp rise after the wedding. Southland's low population density (3.33 people per km^2^) helped officials attribute the spike to this cluster rather than community transmission (Grant, 2008). We apply our model framework to infer parameters and identify the most probable model, anticipating the SSE and SSEB models to be the best fit due to the super-spreading event.

We also analyze the August 2021 SARS-CoV-2 outbreak in Auckland, which coincided with New Zealand's Level 4 lockdown (Department of the Prime Minister and Cabinet, New Zealand, 2024). On August 17th, the government imposed the highest alert level nationwide, but while restrictions were eased elsewhere on August 23rd, Auckland remained under Level 4 (Cumming, 2022). We use case incidence data from Waitemata district of Auckland before and after this date (Fig. 4) and apply our model framework to infer parameters and identify the most probable model.

We applied our model framework to the March 2020 and August 2021 New Zealand outbreaks, inferring parameters for the five models using MCMC. The basic reproduction number, *R*_0_, estimates across models are shown in Table 5 and are generally consistent. For the March 2020 outbreak, the mean *R*_0_ is 2.11 with standard deviation 0.19, and for August 2021, the mean *R*_0_ is 1.92 with standard deviation 0.13. For both outbreaks *R*_0_ is significantly greater than 1.0, consistent with the fact that the outbreaks have not died out in the selected time series.

**Table 5:** The mean and 95 % CI of the estimates of *R*_0_ across the five models when applied to incidence data from the SARS-CoV-2 Outbreaks in New Zealand in 2020 and 2021.

Model	Outbreak, NZ South 2020	Outbreak, Auckland 2021
Baseline	2.30 [1.9, 2.7]	2.0 [1.7, 2.3]
SSE	2.25 [1.18, 3.35]	2.0 [1.3, 2.8]
SSI	1.80 [1.1, 2.9]	1.65 [0.85, 2.55]
SSEB	2.10 [1.2, 2.9]	1.90 [1.32, 2.55]
SSIB	2.10 [1.1, 3.08]	2.0 [1.2, 3.26]

We performed model selection of our five models applied to the incidence data from New Zealand (Table 6 and Fig. 5). Model evidence was computed using the importance sampling estimator from Equation (24), and posterior model probabilities were subsequently computed in order to carry out model selection. For both outbreaks, the SSE model is selected as the most likely, with posterior probabilities of 0.68 and 0.75, followed by the SSEB model at 0.32 and 0.25. These findings align with prior knowledge of super-spreading events in each outbreak; the Bluff wedding in 2020 and a church gathering in Auckland in 2021.

**Table 6:** Posterior model probabilities of the models when fit to the reported cases of the specific regional outbreaks of SARS-CoV-2 in New Zealand in 2020 and 2021.

Model	Outbreak, NZ South 2020	Outbreak, Auckland, NZ 2021
Baseline	0 (−210)	0 (−110)
SSE	0.68 (−56)	0.75 (−66)
SSI	0 (−126)	0 (−75)
SSEB	0.32 (−57)	0.25 (−67)
SSIB	0 (−85)	0 (−140)

The parameter estimates for the best-fitting SSE and SSEB models are displayed in Table 7. For the SSE model, the 2020 Southland outbreak estimates are *R*_0_ = 2.26, (95 % CI; 1.17, 3.37) and *k* = 0.32 (95 % CI; 0.11, 0.57). For the 2021 Auckland outbreak, *R*_0_ = 1.99 (95 % CI; 1.3, 2.8) and *k* = 0.41 (95 % CI; 0.17, 0.7). The low estimates of *k* indicate a high level of over-dispersion in the average number of secondary cases transmitted. James et al. (2021) estimated *k* = 0.29 (95 % CI; 0.10, 0.51) for SARS-CoV-2 in New Zealand using contact tracing data, which aligns with our estimates: *k* = 0.32 (95 % CI; 0.11, 0.57) and *k* = 0.41 (95 % CI; 0.17, 0.70). Notably, we are able to obtain comparable estimates of *k* from incidence data, similar to those obtained by fitting the offspring distribution to secondary case data. For the SSEB model's *α* parameter, estimates are *α* = 0.33 (95 % CI; 0.02, 0.68) and *α* = 0.20 (95 % CI; 0.05, 0.42). Since 1 − *α* reflects the proportion of *R*_0_ from super-spreading events, values of 0.67 and 0.80 suggest most transmission is due to super-spreading rather than homogeneous spread. The super-spreading parameter *β* is estimated at 8.65 (95 % CI: 5.98, 11.61) for 2020 and *β* = 4.67, 95 % CI (2.85, 6.63) for the outbreak in 2021. Our novel bimodal model yields conclusions about super-spreading that align with those of the established SSE model, which strengthens the validity of our approach. This alignment underscores the potential of our models for use in a public health setting to identify the predominant modes of epidemic transmission.

**Table 7:** The model parameter estimates (mean and 95 % CI) of the SSE and SSEB models - models selected with the highest posterior model probabilities - when applied to incidence data from New Zealand in 2020 and 2021.

Model	Parameter	Outbreak, NZ South 2020	Outbreak, Auckland, NZ 2021
SSE	*R*_0_	2.25 [1.18, 3.35]	2.0 [1.3, 2.8]
	*k*	0.32 [0.11, 0.57]	0.41 [0.17, 0.7]
SSEB	*R*_0_	2.10 [1.2, 2.9]	1.9 [1.32, 2.55]
	*α*	0.33 [0.02, 0.68]	0.2 [0.01, 0.42]
	*β*	8.65 [5.98, 11.61]	4.67 [2.85, 6.63]

### 3.4 SARS Outbreak, Canada 2003

We apply our modelling framework to the outbreak of SARS in Canada in 2003. Severe Acute Respiratory Syndrome (SARS) is a viral respiratory illness caused by a coronavirus. The SARS outbreak originated in Asia in late 2002 and spread internationally. Canada's first case was reported in Toronto, Ontario, on February 23rd, 2003 (Varia et al., 2003). The initial infection was traced back to a woman who had travelled to Hong Kong and returned to Toronto, resulting in a large nosocomial outbreak in a Toronto hospital, that yielded 128 infections (Varia et al., 2003). We speculate that this individual could be a super-spreading individual. For our analysis we focus on two waves of infection that see a spike in the number of reported cases in Canada (Fig. 6). For the outbreak of SARS in 2003 a serial interval distribution with a median of 6 days and an inter-quartile range of 4–9 days was previously reported (Lloyd-Smith et al., 2005). We therefore use a discretised Gamma(6,1) as the generation time distribution. The *R*_0_ estimates derived from MCMC are generally consistent across the five models, with low standard deviation observed for all models. For wave 1 the mean estimate of *R*_0_ across the five models is 1.36 with standard deviation 0.04. For wave 2, the mean estimate of *R*_0_ is 1.82 with standard deviation 0.96.

For both waves the super-spreading infections models emerge as the most likely models, specifically the SSIB model followed by the SSI model (Fig. 6). The SSIB model has a posterior model probability of 0.77 and 0.88 for the two waves respectively. The SSI model has the second highest posterior model probabilities of 0.23 and 0.20. The results align with reports in the literature that an individual caused a large nosocomial outbreak in a Toronto hospital, resulting in 128 infections (Varia et al., 2003). This suggests the presence of super-spreading individuals, as indicating by our model selection process. For the SSI model, *R*_0_ = 1.37 (95 % CI; 1.96, 2.0) for wave 1 and *R*_0_ = 1.75 (95 % CI; 1.2, 2.3) for wave 2, with dispersion parameter *k* = 0.27 (95 % CI; 0.08, 0.51) for wave 1 and *k* = 0.30 (95 % CI; 0.05, 0.62) for wave 2, indicating over-dispersion in secondary cases. For the SSIB model, *R*_0_ = 1.30 (95 % CI; 1.04, 2.0) for wave 1 and *R*_0_ = 1.85 (95 % CI; 1.3, 3.0) for wave 2. The parameter *a* = 0.37 (95 % CI; 0.05, 0.76) for wave 1 and *a* = 0.37 (95 % CI; 0.23, 0.53) for wave 2 further suggests that transmission is predominantly driven by super-spreading rather than homogeneous transmission.

## 4 Discussion

The primary aim of this work is to develop a modelling framework for incidence time-series data that encompasses distinct models for super-spreading events and super-spreading individuals. Additionally, it seeks to perform model selection between these candidate models when fit to both simulated and, more critically, real epidemic data. To achieve this, a comprehensive framework of stochastic branching process models of epidemic transmission is developed for time-series of incidence data that encompasses five distinct models. The SSEB and SSIB models presented introduce novel bimodal approaches to characterize super-spreading events and individuals. The models include distinct mechanisms of epidemic transmission.

We have successfully implemented MCMC algorithms to infer the parameters of all five models. The results of our quantitative inference study highlight the effectiveness of the inference methods. This is particularly true for the basic reproduction number *R*_0_ across all models and is also supported when we apply our methods to incidence time-series of real outbreak data. Our estimates of *k* align closely with those obtained in studies using secondary case data, underscoring the utility of incidence data, which is more widely available. For example, while some studies rely on detailed contact tracing to estimate transmission dynamics (James et al., 2021), our models achieve similar insights using temporal data alone. This capability could significantly streamline surveillance during outbreaks by reducing reliance on resource-intensive data collection. We can also infer the novel parameters of our novel super-spreading models, namely *α* and *β* of the SSEB model and *a* and *b* in the SSIB model; when the super-spreading infections data is available. Interestingly when we compare the estimates of our best fitting SSE and SSEB models to the outbreak of SARS-CoV-2 in New Zealand for example, we observe low values of both *k* and *α* in the SSE and SSEB models respectively. In the definition of our model this corresponds to a large amount of super-spreading or over-dispersion in both cases. Similarly for the outbreak of SARS in Canada in 2003 we estimate low values for both *k* and *a* in the best fitting SSI and SSIB models. Such results provide support for our SSEB and SSIB models as novel methods for quantifying super-spreading.

Model comparison is an important feature of our framework. Across simulations, the true simulation model consistently achieves the highest posterior probability, confirming that the five models are both identifiable and capable of capturing distinct mechanisms of epidemic transmission. When tested with real outbreak data, the model selection results exhibit clear and consistent trends. We extract time-series of distinct time periods of each disease and region. Yet the same models are consistently selected as the best fit for the same disease and region at different points in time, while different models are selected for different diseases. This suggests that the mechanisms of epidemic transmission of some of our models are a better fit to certain diseases than others.

For SARS-CoV-2, which caused the major Covid-19 pandemic, the SSE and SSEB models, which account for super-spreading events, provided the best fit. This aligns with the observed epidemiology of SARS-CoV-2, where super-spreading events played a significant role in transmission dynamics (Brainard et al., 2023; Du et al., 2022; Lewis, 2021). In contrast, the SARS outbreak in 2003, which was controlled to an extent and did not escalate into a global pandemic, was best described by the SSI and SSIB models. These models emphasize individual-level heterogeneity. Notably the Baseline Poisson model, that assumes homogeneous transmission in the population with no capacity for over-dispersion, i.e. equal mean and variance, is never selected as the best fitting model. This provides further support for the necessity of incorporating dispersion or super-spreading in models for epidemic transmission.

From an epidemiological standpoint, the simplicity of the models, with a maximum of three parameters, is both a strength and a limitation. While the simplicity aids in the clarity and applicability of the models, the models may not capture the full extent of the complexities of real-world epidemics where super-spreading events and individuals often occur simultaneously. A more complex model combining both SSE and SSI could potentially provide a more accurate representation. However there are benefits to keeping models as simple as possible, such as their rapid implementation and flexibility across use-cases. We only consider constant parameters, we do not consider variations of our parameters over time, for example a time-varying reproduction number *R*_*t*_ (Cori et al., 2013) or time-varying *k*_*t*_ (Adam et al., 2022; Ho et al., 2023). This limitation means that our models may not capture the dynamic nature of epidemic spread, where parameters can change over time due to factors such as public health interventions, changes in population behavior, or pathogen evolution. However, we focus on relatively short time windows, which somewhat mitigates this limitation. Additionally, the assumption of complete reporting of infectious cases overlooks potential sampling biases in real-world data. While this is a common assumption in the literature (Cori et al., 2013), it remains a potential source of inaccuracy. A potential solution to this would be incorporating the sampling proportion as a parameter in our models. However, considering a partially observed branching process can be complicated. It requires accounting for the possibility that entire sections of a transmission tree, which depicts the chain of transmission events within a population, may be observed or missed. This complexity arises because the available data might not capture every individual case or transmission event, making it challenging to accurately reconstruct the full transmission dynamics (Didelot et al., 2017; Carson et al., 2024).

In conclusion, this work presents a novel modelling framework using time series incidence data to analyze super-spreading phenomena, offering an alternative to models that rely on less accessible secondary case data. We developed and validated five distinct stochastic branching-process models, alongside a robust comparison method that differentiates them, capturing unique epidemic transmission mechanisms. Our approach consistently identifies transmission mechanisms across different time series, highlighting the inadequacy of homogeneous transmission assumptions and emphasizing the need for models that incorporate dispersion and super-spreading. Quantifying the impact of super-spreading is crucial for disease control; focusing efforts on super-spreading events could significantly reduce the reproduction number, as previously noted (Endo, Abbott, Kucharski, & Funk, 2020). Public health initiatives can leverage our models to target super-spreading events and individuals for tailored interventions, as seen in Japan during the COVID-19 pandemic (Ueda et al., 2023). This targeted approach allows for efficient resource allocation, potentially curbing epidemic spread more effectively than generalized strategies. Overall, our modelling framework provides valuable insights for public health officials, enhancing epidemic management and prevention.

## CRediT authorship contribution statement

**Hannah Craddock:** Writing – review & editing, Writing – original draft, Software, Methodology, Investigation, Conceptualization. **Simon E.F. Spencer:** Writing – review & editing, Writing – original draft, Methodology, Investigation, Conceptualization. **Xavier Didelot:** Writing – review & editing, Writing – original draft, Methodology, Investigation, Conceptualization.

## Declaration of competing interest

The authors declare that they have no known competing financial interests or personal relationships that could have appeared to influence the work reported in this paper.
