# Supplementary material for: A Bayesian modelling framework with model comparison for epidemics with super-spreading
Source: Infect Dis Model. 2025 Aug 5;10(4):1418–32. doi: 10.1016/j.idm.2025.07.017 (PMC12351329; doi:10.1016/j.idm.2025.07.017)
Supplement: Multimedia component 1 [file mmc1.pdf]

Supplementary Material

June 27, 2025

Contents

Page

1 Background **2**
1.1 Summary of Epidemiological Parameter Estimates 2

2 Modelling Framework – Additional Details of Model Derivations **4**
2.0.1 Individual Level Infectiousness 4
2.0.2 Population Level Infectiousness across Time 5
2.0.3 Additional Notation for Model Derivations 5
2.1 The Baseline Model 6
2.1.1 The Offspring Distribution 6
2.1.2 The Incidence Model 6
2.1.3 Derivation of Z from $\boldsymbol{I}_{[1:T]}$ 7
2.2 The SSE Model 7
2.2.1 The Offspring Distribution 7
2.2.2 The Incidence Model 8
2.2.3 Derivation of Z from $\boldsymbol{I}_{[1:T]}$ 8

3 Prior Selection for Bayesian Inference **10**
3.1 Prior Distributions of the Model Parameters 12

4 Prior Sensitivity Analysis – Parameter Inference **13**
4.1 Prior Sensitivity Analysis – R_0 13

5 Model Selection Simulation Study Results **15**
5.1 Results and Interpretation 16
5.1.1 Baseline Simulation Model 18
5.1.2 SSE Simulation Model 19
5.1.3 SSI Simulation Model 20
5.1.4 SSEB Simulation Model 21
5.1.5 SSIB Simulation Model 22

1 Background

1.1 Summary of Epidemiological Parameter Estimates

We provide a summary of estimates from various modelling studies of key epidemiological parameters R_0 and k estimated for a number of different diseases in Table I. These estimates help to inform our selection of priors for Bayesian inference and also guide the parameter values used in our simulation studies.

It is important to note that these values should be interpreted with caution. As discussed in Delamater et al. (2019), such parameter values are derived from mathematical models, and estimates are influenced by various choices made during the modeling process. Comparing the contagiousness of different historical, emerging, and reemerging infectious agents is not entirely accurate unless recalculated using consistent modeling assumptions. Consequently, some of the R_0 values frequently cited in the literature for past epidemics may not be applicable to current outbreaks of the same infectious diseases. Nevertheless R_0 and k are highly valuable metrics of disease transmission.

Parameter	Disease (Variant)	Mean/Median & CI, CrI, IQR	Method or Model	Reference
$\boldsymbol{R_0}$	**Ebola** (2014)	1.8 (Median), IQR; (1.4 - 1.8)	Pooled estimate, systematic review of 29 studies	Wong et al. (2017)
	**Influenza** (Seasonal)	1.28 (Median), IQR; (1.19 - 1.37)	Pooled estimate, systematic review of 24 studies	Biggerstaff et al. (2014)
	**Influenza** (2009; A/H1N1)	1.46 (Median), IQR; (1.3 - 1.7)	Pooled estimate, systematic review of 57 studies	Biggerstaff et al. (2014)
	**Influenza** (Avian; H5N1, H5N8, H5N6)	1.69 (Mean), 95% CI (1.48, 2.39)	SIR model	Kim and Cho (2021)
	**MERS-CoV** (2015)	0.47 (Mean), 95 % CI (0.29, 0.89)	$Z \sim \text{NegBin}(\mu = R_0, k)$	Kucharski and Althaus (2015)
	**Monkeypox**	2.1 (Mean), 95 % CI: (1.1, 2.7)	SEIR model	Grant et al. (2020)
	**SARS** (Hong Kong, 2003)	2.7 (Mean), 95 % CI (2.2, 3.7)	Stochastic compartmental model fit to incidence time series	Riley et al. (2003)
	**SARS-CoV-2** (Early phase)	1.23 (Mean), 95% CI (1.09, 1.39)	$Z \sim \text{NegBin}(R_0, k)$	Wang et al. (2020)
	**SARS-CoV-2** (alpha)	3.28 (Mean), Range: 1.4 -6.5	Pooled estimate of systmatic review	Liu et al. (2020)
	**SARS-CoV-2** (alpha)	2.5 (Mean), 95% CI: (2-3)	$Z \sim \text{NegBin}(R_0, k)$	Endo et al. (2020)
$\boldsymbol{k}$	**SARS-CoV-2** (Early phase)	0.23 (Mean), 95 % CI: (0.13, 0.38)	$Z \sim \text{NegBin}(R_0, k)$	Wang et al. (2020)
	**SARS-CoV-2** (Early phase, New Zealand)	0.29 (0.10, 0.205)	$Z \sim \text{NegBin}(R_0, k)$	James et al. (2021)
	**SARS-CoV-2** (alpha)	0.1 (Mean), 95 % CI: (0.05, 0.2)	$Z \sim \text{NegBin}(R_0, k)$	Endo et al. (2020)
	**SARS-CoV-2** (alpha, Hong Kong)	0.20 (Mean), 95 % CI (0.16, 0.25)	$Z \sim \text{NegBin}(R_0, k)$	Adam et al. (2022)
	**SARS** (Hong Kong, 2003)	0.04 (Mean), 95 % CI (0.03, 0.06)	$Z \sim \text{NegBin}(R_0, k)$	Adam et al. (2022)
	**MERS-CoV** (2015)	0.16 (Mean), 95 % CI (0.11, 0.64)	$Z \sim \text{NegBin}(R_0, k)$	Kucharski and Althaus (2015)

Table I: Estimates in the literature of epidemic parameters, R_0 and k from models fit to real epidemic outbreak data of the listed diseases and their variants. The mean or median and credible intervals (CI), confidence intervals (CI) or Interquartile range of the estimates are listed.

2 Modelling Framework – Additional Details of Model Derivations

To generate incidence data I_t at time t from each of the five models we require knowledge of the infectious pressure from individuals infected at earlier time-points. We first consider the infectious contribution of one infected individual over their infectious period. We then show how this is incorporated for all infections at the population level for the duration of the epidemic. The fact that the models are branching process models means that all individuals have the same offspring distribution allowing us to move from the individual level to the population level and vice versa.

2.0.1 Individual Level Infectiousness

For the infectious contribution of one infection or infected individual, we make the assumption that each new infection has an infectivity profile given by a probability distribution denoted $\omega(\tau)$, dependent on the time since infection, time τ. A similar approach of using the infectivity profile is seen in Cori et al. (2013). As outlined in Cori et al. (2013) an individual will be most infectious at time τ when $\omega(\tau)$ is at its maximum. Throughout our study we use a discretized gamma function to represent the infectivity profile given that the incidence data is in discrete time. The discretized gamma is implemented using the cumulative distribution function (CDF) and parameterised with shape θ and scale σ as follows;

$$\omega(\tau) = F_{\text{Gamma}}(\tau; \theta, \sigma) - F_{\text{Gamma}}(\tau - 1; \theta, \sigma) \tag{1}$$

This infectivity sums to one over the infectious lifetime of a single infected individual, i.e;

$$\sum_{\tau=1}^{\infty} \omega(\tau) = 1 \tag{2}$$

For this work we select a Gamma($\theta = 6$, $\sigma = 1$) distribution to represent the infectivity profile as illustrated in Figure I. The Gamma($\theta = 6$, $\sigma = 1$) distribution has a mean of shape$\times$scale = 6, a mode of 5 and standard deviation of 2.45. Such summary statistics align with estimates of the serial interval distribution for diseases such as SARS-CoV-2 and SARS as discussed in relation to applications to real data in the main paper. To illustrate Equation 2, consider the sum from $\tau = 1$ to ∞, approximated by setting ∞ to 1000. For $\omega(\tau)$ defined in Equation 1, this sum equals 1 for the gamma distribution with shape = 6 and scale = 1.

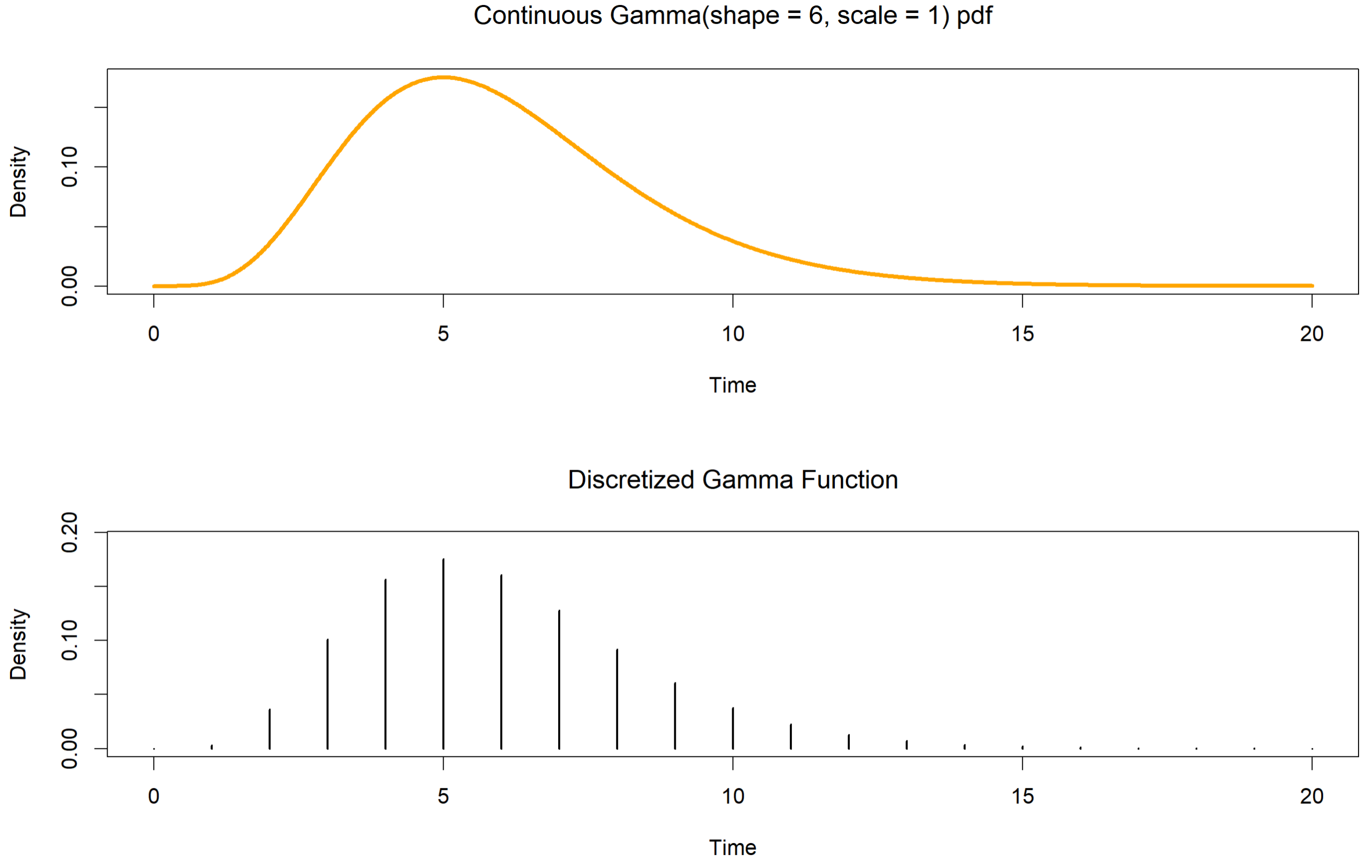

Figure I: The Discretized gamma function, Gamma(shape $=6$, scale $=1$) used to represent the infectivity profile of a single individual is shown in the bottom plot. This distribution has a mean of shape $\times$ scale $=6$, a mode of 5 and $\sigma = 2.45$. The continuous Gamma(shape $=6$, scale $=1$) pdf is shown in the top plot however it is not directly used

2.0.2 Population Level Infectiousness across Time

To generate incidence data I_t at time t for each of the five models, it is necessary to account for the infectious pressure from individuals who were infected at previous time points. We define λ_t as the total infectious pressure of the population as in the main paper and it is a function of all five models.

$$\lambda_t = \sum_{\tau=1}^{t-1} I_\tau \cdot \omega(t-\tau), \qquad \text{for } t = 2 \dots T. \tag{3}$$

2.0.3 Additional Notation for Model Derivations

For our model derivations we introduce a variable Ω which is the cumulative number of people infected between time $t=2$ and $t=T$ and is therefore the sum of infections over this

time period. As the infections generated at time $t = 2$ depend on the infectious contribution from infections at time $t = 1$ we have;

$$\Omega \mid I_1 = \sum_{t=2}^{T} I_t \tag{4}$$

For short-hand for are derivations we will refer to $\Omega \mid I_1$ as Ω.

We next provide additional details of the model derivations for the Baseline model and the SSE model.

2.1 The Baseline Model

In the Baseline model, the offspring distribution Z and the incidence data I_t follow Poisson distributions.

2.1.1 The Offspring Distribution

In this model the offspring distribution of a single individual is defined to be

$$Z \sim \text{Poisson}(R_0) \tag{5}$$

The mean of a Poisson distribution is equal to its parameter and as before

$$\mathbb{E}[Z] = R_0. \tag{6}$$

2.1.2 The Incidence Model

We now detail how the incidence data is derived from the Baseline model. As the model is a branching process model, all individuals have the same offspring distribution and we can use this fact to construct the incidence data I_t, the number of infections at time t. For the Baseline model we define I_t as the number of infections at time t as a Poisson random variable with mean $R_0 \cdot \lambda_t$. The parameter λ_t as outlined in Equation 3 is the total infectious pressure of the population which we infer from the the infectivity profile $\omega(\tau)$ and the incidence data up to the previous time point; $\boldsymbol{I}_{[1:t-1]}$. The incidence data from the Baseline model is

$$I_t \sim \text{Poisson}(R_0 \cdot \lambda_t) \tag{7}$$

Recall that the total infectious pressure λ_t is defined as

$$\lambda_t = \sum_{\tau=1}^{t-1} I_\tau \cdot \omega(t - \tau), \qquad \text{for } t = 2 \ldots T. \tag{8}$$

In the baseline model, the basic reproduction number R_0 is the single parameter of inference.

2.1.3 Derivation of Z from $\boldsymbol{I}_{[1:T]}$

We next demonstrate how we can re-derive the offspring distribution Z of the Baseline model as in Equation 5 from the incidence data in Equation 7. We introduce Ω in Equation 4 as the cumulative number of infections during the epidemic and therefore the sum of all infections up to time T. For the Baseline model, this is the sum of individual Poisson distributions which is itself a Poisson distribution;

$$\Omega \sim \text{Poisson}(R_0\lambda_2) + \text{Poisson}(R_0\lambda_3) + \ldots + \text{Poisson}(R_0\lambda_T) \tag{9}$$

$$\Omega \sim \text{Poisson}\left(\sum_{t=2}^{T} R_0\lambda_t\right) \tag{10}$$

For the offspring distribution Z we are interested in the cumulative number of infection cases due to one infected individual and their infectious contribution over their entire infectious lifetime. From Equation 2 we have that the infectivity of one individual over their entire infectious duration sums to one and so

$$\lim_{T\to\infty} \sum_{t=2}^{T} \lambda_t = \lim_{T\to\infty} \sum_{t=2}^{T} \sum_{\tau=1}^{t-1} I_\tau \cdot \omega(t-\tau) \tag{11}$$

$$\text{For one infected individual, this simplifies to} \tag{12}$$

$$= \lim_{T\to\infty} \sum_{t=2}^{T} 1 \cdot \omega(t-1) \tag{13}$$

$$\text{Now referring to Equation 2 and applying a change of variable} \tag{14}$$

$$= \sum_{u=1}^{\infty} \omega(u) \tag{15}$$

$$= 1 \tag{16}$$

Substituting Equation 16 into Equation 10 gives the offspring distribution Z of one case as

$$Z \sim \text{Poisson}(R_0) \tag{17}$$

2.2 The SSE Model

In the SSE model, the offspring distribution Z and the incidence data I_t follow negative binomial distributions.

2.2.1 The Offspring Distribution

In the SSE model the offspring distribution of a single individual is defined to be

$$Z \sim \text{Negative Binomial}(k, \mu = R_0) \tag{18}$$

The mean of the offspring distribution Z is equal to μ and it follows trivially that

$$\mathbb{E}[Z] = R_0. \tag{19}$$

This parameterisation is analogous to the negative binomial model with parameters k and probability $p = \frac{k}{\mu+k}$ as follows;

$$Z \sim \text{Negative Binomial}(k, p = \frac{k}{R_0 + k}) \tag{20}$$

The Variance of Z is $\frac{R_0(R_0+k)}{k}$. As $k \to 0$, Var $\to \infty$ and therefore small values of k are indicative of increased heterogeneity in transmission.

2.2.2 The Incidence Model

We now outline how the incidence data I_t is derived from the SSE model. The models are branching process models and so all individuals have the same offspring distribution. We can use this fact to construct the incidence data I_t. For the SSE model we define I_t as the number of infections at time t as a negative binomial random variable parameterised with size r and mean μ and model parameters R_0 and k. We use an analogous parameterisation to that of Ho et al. (2023) however they incorporate the time-varying reproduction number R_t instead of the basic reproduction number R_0. In our SSE model the incidence data is defined as

$$I_t \sim \text{Negative Binomial}\left(r = k\lambda_t, \;\; \mu = R_0\lambda_t\right) \tag{21}$$

2.2.3 Derivation of Z from $\boldsymbol{I}_{[1:T]}$

We next show that we can re-derive the offspring distribution Z in the SSE model as in Equation 18 from the incidence data $\boldsymbol{I}_{[1:T]}$ as in Equation 21. As defined in Equation 4, Ω is the cumulative number of infections from $t = 2$ up to T and therefore the sum of all infections during this time period. For the SSE model this is the sum of individual negative binomial distributions. Using the parameterisation of the negative binomial distribution with size r and probability p, we can exploit the fact that the sum of independent, negative binomial (NB) random variables with the same probability parameter p is also a negative binomial distribution with probability parameter p;

$$\Omega \sim \text{NB}(r_1, p) + \text{NB}(r_2, p) + \cdots + \text{NB}(r_T, p) = \text{Negative Binomial}\left(\sum_{t=2}^{T} r_t, p\right) \tag{22}$$

For the specific parameters of the SSE model and this parameterisation of the negative binomial distribution with size r and probability p, Equation 22 becomes

$$\Omega \sim \text{Negative Binomial}\left(r = \sum_{t=2}^{T} k\lambda_t, p = \frac{k}{R_0 + k}\right). \tag{23}$$

For the offspring distribution Z we are interested in the cumulative number of infection cases due to one infected individual and their infectious over their entire infectious duration. From Equation 2 we have that their infectivity over their entire infectious duration sums to one and so

$$\lim_{T\to\infty}\sum_{t=2}^{T}\lambda_t = \lim_{T\to\infty}\sum_{t=2}^{T}\sum_{\tau=1}^{t-1} I_\tau \cdot \omega(t-\tau) \tag{24}$$

$$\text{For one infected individual, this simplifies to} \tag{25}$$

$$= \lim_{T\to\infty}\sum_{t=2}^{T} 1 \cdot \omega(t-1) \tag{26}$$

$$\text{Now referring to Equation 2 and applying a change of variable} \tag{27}$$

$$= \sum_{u=1}^{\infty}\omega(u) \tag{28}$$

$$= 1 \tag{29}$$

Substituting Equation 29 into Equation 23 gives the offspring distribution Z of one single case as

$$Z \sim \text{Negative Binomial}\left(k, p = \frac{k}{R_0 + k}\right). \tag{30}$$

The equivalent parameterisation using k and μ is

$$Z \sim \text{Negative Binomial}\left(k, \mu = R_0\right). \tag{31}$$

3 Prior Selection for Bayesian Inference

In our Bayesian framework for model inference, we primarily opt for weakly informative priors to guide our analysis. These priors allow a balance between prior knowledge and the data-driven inference process. This helps to avoid extreme estimates and overly constraining the results. Our framework remains disease-agnostic, allowing for the selection of more informative priors tailored to specific datasets as required. The prior distributions chosen for our models are displayed in Table II and can be summarised as follows

- For the parameter R_0, common to all five models, an Exponential(1) distribution is used as the prior distribution throughout the study. This distribution with a mean centered on 1 implies that a priori, we are not specifying whether the outbreak is likely to propagate throughout the population, $R_0 > 1$, or die out, $R_0 < 1$. The Exponential(1) distribution has 95% quantiles $[0.025, 3.688]$. Referring to estimates of R_0 of real, recent infectious disease epidemics (see Table I), this interval covers the full range of the estimates and their respective credible intervals. The Exponential(1) prior on R_0 is also compared to a Gamma(1,5) prior and a Uniform(0,10) prior in our prior sensitivity analysis

- For the dispersion parameter k in the SSE and SSI models, an Exponential(5) prior is used. This prior has a mean of 0.2 and a 95% quantiles ; [0.051,0.693]. This range encompasses the credible intervals of k fit to SARS-CoV-2 data, which Endo et al. (2020) found to be [0.05-0.2]. The range also aligns with the estimate and credible intervals of k fit to the MERS outbreak in 2015; 0.16 [0.11, 0.64] (see Table I).

- For the proportion parameters α and a in the SSEB and SSIB models respectively, we select a Beta$(2, 2)$ prior distribution. This distribution has the requisite restricted support of $[0, 1]$ and a 95% quantiles that spans the range [0.035, 0.965]. The parameters α and a represent the proportion of R_0 due to non super-spreading and a priori, we want there to be equal prior probability on the incidence of no super-spreading versus super-spreading. Therefore we choose a prior distribution with mean centred on 0.5. A further motivation for using this prior is to do with the nested model structure and will become important for model comparison. Recall that the models SSEB and SSIB are nested within the Baseline model, i.e they reduce to the Baseline model if $\alpha = 1$ and $a = 1$. Johnson and Rossell (2010) highlights that the prior densities used to define alternative hypotheses in Bayesian tests, for example no super-spreading versus superspreading, often assign non-negligible probability to regions within the parameter domain that correspond to the null hypothesis. Hence apriori we are assigning minimal probability to this occurrence by using the Beta$(2, 2)$ which has negligible mass close to 0 or 1. A prior sensitivity analysis is conducted for the parameter α in which we compare the results of inference using a Uniform(0,1) prior

- For the super-spreading parameter β of the SSEB model, which represents the increased factor of infections due to a super-spreading event, we choose a shifted Gamma distribution. The chosen prior is a 1 + Gamma(3,3) which has a mean of 10 and a 95 % quantiles that spans the range [2.9, 22.7]. Super-spreading events are events that

result in a larger than average number of infections. We select a shifted Gamma to ensure that the prior value for β is always greater then one, to capture our prior belief regarding super-spreading events. A prior sensitivity analysis is also conducted for the parameter β in which we compare the results of inference using a Uniform(1,40) prior

- For the super-spreading parameter b of the SSIB model, which represents the increased infectivity of SSIs, we also choose a shifted $1 + \text{Gamma}(3, 3)$ as the prior distribution. Given that b is a super-spreading parameter, our prior assumption is that this value is greater than 1, which is why we introduce a shift of 1 in the prior distribution. Wallinga and Teunis (2004) defines a super-spreader as one that yields at least 10 secondary infections. Therefore a prior with a mean of 10 and quantiles that span a moderately wide range is selected

3.1 Prior Distributions of the Model Parameters

**Model**	**Model Distribution**	**Parameter**	**Prior Distribution**	**Prior support**	**Prior Mean**	**Prior 95 % Quantiles**
**Baseline**	Poisson	$\boldsymbol{R_0}$	Exponential(1)	$[0, \infty]$	1	[0.025, 3.7]
**SSE**	Negative Binomial	$\boldsymbol{R_0}$	Exponential(1)	$[0, \infty]$	1	[0.025, 3.7]
		$\boldsymbol{k}$	Exponential(5)	$[0, \infty]$	0.2	[0.05, 0.7]
**SSI**	Poisson(Gamma())	$\boldsymbol{R_0}$	Exponential(1)	$[0, \infty]$	1	[0.025, 3.7]
		$\boldsymbol{k}$	Exponential(5)	$[0, \infty]$	0.2	[0.05, 0.7]
**SSEB**	Compound Poisson	$\boldsymbol{R_0}$	Exponential(1)	$[0, \infty]$	1	[0.025, 3.7]
		$\boldsymbol{\alpha}$	Beta(2,2)	[0, 1]	0.5	[0.035, 0.965]
		$\boldsymbol{\beta}$	1 + Gamma(3,3)	$[1, \infty]$	10	[2.9, 22.7]
**SSIB**	Compound Poisson	$\boldsymbol{R_0}$	Exponential(1)	$[0, \infty]$	1	[0.025, 3.7]
		$\boldsymbol{a}$	Beta(2,2)	[0, 1]	0.5	[0.035, 0.965]
		$\boldsymbol{b}$	1 + Gamma(3,3)	$[1, \infty]$	10	[2.9, 22.7]

Table II: The model parameters, their chosen prior distributions and the relevant metrics of the chosen distributions including the support, mean and 95 % quantiles. Further details regarding our choice of prior distributions is available in the Supplementary materials

4 Prior Sensitivity Analysis – Parameter Inference

A prior sensitivity analysis is conducted to evaluate the impact of the choice of prior on Bayesian inference of the model parameters. The analysis is important to ensure that the outcomes of Bayesian inference are not unduly influenced by specific prior choices. For this research we focused our prior sensitivity analysis on the parameter R_0, as it is a parameter common to all five models. In addition we evaluated the prior sensitivity of the super-spreading parameters in Craddock (2024).

4.1 Prior Sensitivity Analysis – R_0

For the parameter R_0, common to all five models, an Exponential(1) distribution is used as the prior distribution throughout the study as outlined in section 3. For our prior sensitivity analysis we trialled two additional priors on R_0; a Gamma(1,5) prior following the work of Cori et al. (2013) and a Uniform(0,10) prior as its support encompasses a large range of possible values of R_0. We evaluated the estimates of our posterior distributions of R_0 using these priors over a large number of repetitions, $n = 3000$, similar to our earlier quantitative analyses. The prior sensitivity analysis for R_0 is conducted for both the Baseline and SSE models. Incidence data $\boldsymbol{I}_{[1:T]}$ is simulated with R_0 within $[1, 2]$, $[2, 3]$, and $[3, 4]$ ($n = 1000$ in each interval) from both models. For the Baseline model, we infer the posterior distributions of R_0 using MCMC (Metropolis Hastings algorithm) with three different priors on R_0: Exponential(1), Gamma(1,5), and Uniform(0,10). We repeat the same process for the SSE model, inferring the posterior distributions of R_0 using our adaptive shaping and scaling algorithm, testing the the same three priors. The results of the Baseline model analysis are displayed in Table III and for the SSE model in Table IV.

Results

The sensitivity analysis of R_0 indicates that the prior choice has little influence on its Bayesian inference. The results show little variation across the three priors, indicating that Bayesian inference is predominantly influenced by the likelihood and the data. For both the Baseline model results (Table III) and the SSE model results (Table IV), there is little discrepancy in the biases or 95% credible intervals of the estimates between the three priors. For instance, the bias for the Exponential(1) prior for R_0 in the interval [3, 4] is is 0.002. For the Gamma(1,5) prior, this was -0.005, and for the Uniform(0,10) prior, -0.007. The coverage is high for all three priors, with 96%, 96%, and 95% respectively for R_0 in the range [1,2]. We have already shown from our quantitative analysis that inference of R_0 is highly effective across all five models. This prior sensitivity analysis provides further evidence that our Bayesian inference methods for the parameter R_0 are robust and reliable regardless of the choice of prior.

	**Baseline model** – R_0		
	**Exponential(1) Prior on** R_0	**Gamma(1, 5) Prior on** R_0	**Uniform(0, 10) Prior on** R_0
**Prior Mean & 95 % CI**	**1 [0.025, 3.7]**	**5 [1.7, 11.15]**	**5 [0.25, 9.75]**
$\boldsymbol{R_0}$ **Simulated: [1, 2]**			
Bias	-0.01	0.01	0.04
95 % CI (mean)	[1.36, 1.84]	[1.13, 2.10]	[1.12, 2.18]
Coverage (%) (mean)	96	96	95
$\boldsymbol{R_0}$ **Simulated: [2, 3]**			
Bias (mean)	-0.02	-0.06	-0.03
95 % CI (mean)	[2.22, 2.70]	[2.23, 2.73]	[2.26, 2.74]
Coverage (%) (mean)	93	93	94
$\boldsymbol{R_0}$ **Simulated: [3, 4]**			
Bias (mean)	-0.002	-0.005	-0.007
95 % CI (mean)	[3.45, 3.53]	[3.36, 3.65]	[3.36, 3.66]
Coverage (%) (mean)	95	94	94

Table III: Prior Sensitivity Analysis of R_0 in the Baseline model. A comparative analysis of the summary statistics of the inferred posterior distributions of R_0 using an Exponential(1), Gamma(1,5) and Uniform(0,10), $n = 1000$ repetitions for each interval.

	**SSE model** – R_0		
	**Exponential(1) Prior on** R_0	**Gamma(1, 5) Prior on** R_0	**Uniform(0, 10) Prior on** R_0
**Prior Mean & 95 % CI**	**1 [0.025, 3.7]**	**5 [1.7, 11.15]**	**5 [0.25, 9.75]**
$\boldsymbol{R_0}$ **Simulated:[1, 2]**			
Bias (mean)	-0.01	0.02	0.05
95 % CI (mean)	[1.23, 2.0]	[1.10, 2.15]	[1.09, 2.20]
Coverage (%) (mean)	96	95	95
$\boldsymbol{R_0}$ **Simulated: [2, 3]**			
Bias (mean)	-0.05	-0.06	-0.04
95 % CI (mean)	[2.22, 2.70]	[2.22, 2.75]	[2.20, 2.78]
Coverage (%) (mean)	93	92	93
$\boldsymbol{R_0}$ **Simulated: [3, 4]**			
Bias (mean)	-0.01	-0.02	-0.02
95 % CI (mean)	[3.37, 3.60]	[3.35, 3.70]	[3.30, 3.72]
Coverage (%) (mean)	94	94	93

Table IV: Prior Sensitivity Analysis of R_0 in the SSE model. A comparative analysis of the summary statistics of the inferred posterior distributions of R_0 using an Exponential(1), Gamma(1,5) and Uniform(0,10), $n = 1000$ repetitions for each interval.

5 Model Selection Simulation Study Results

To validate our model comparison methodology, we begin with a simulation study using data generated from each of our five models before applying the approach to real data. The objective is to assess how consistently model selection identifies the true data-generating model as the most probable among the five candidates.

Simulation Evaluation Framework & Repeated Trials

In this supplementary materials we conduct the model selection simulation study using the selected priors as in section 3. In Craddock (2024) we repeat the analysis across multiple simulation scenarios. We firstly repeat the analysis using Uniform priors across all parameters, like those used in the parameter sensitivity analysis. For both sets of priors we repeat the analyses for $R_0 = 1.5, 2.0$ and 3.0 in our simulation, for epidemics of length 50 days. We also repeat the analyses for epidemics of length 20 days and 100 days.

	**Model comparison evaluation & sensitivity analysis**	
	**Priors used inc Exponential(1) Prior on R_0**	**Uniform priors**
Num days = 50		
$\boldsymbol{R_0}$	1.5	1.5
	2.0	2.0
	3.0	3.0
Num days = 20		
$\boldsymbol{R_0}$	[1.5, 3.0]	[1.5, 3.0]
Num days = 100		
$\boldsymbol{R_0}$	[1.5, 3.0]	[1.5, 3.0]

Table V: A summary of the prior sensitivity analysis undertaken in relation to model selection. The original priors used were compared against Uniform priors on all parameters

Simulation Procedure

For each simulation scenario, we conduct five independent model selection experiments. In each experiment, one model is designated as the data-generating model. We simulate 100 synthetic incidence datasets from this model, then fit all five models to each dataset and compute the posterior model probabilities. The objective is to determine whether the model used for simulation is identified as the most probable model. For instance, when the Baseline model is the data-generating model, we simulate 100 datasets from this model, denoted as $\boldsymbol{I}_{\{[1:T],\text{Baseline}\}}$. For each simulated dataset, we estimate the model evidence and compute the posterior probabilities for each model, i.e; $P(M_{\text{Baseline}} \mid \boldsymbol{I}_{\{[1:T],\text{Baseline}\}})$, $P(M_{\text{SSE}} \mid \boldsymbol{I}_{\{[1:T],\text{Baseline}\}})$, $P(M_{\text{SSI}} \mid \boldsymbol{I}_{\{[1:T],\text{Baseline}\}})$, $P(M_{\text{SSEB}} \mid \boldsymbol{I}_{\{[1:T],\text{Baseline}\}})$, and $P(M_{\text{SSIB}} \mid$

$\boldsymbol{I}_{\{[1:T],\text{Baseline}\}}$). We then assess whether the true model—used for simulation—is most frequently the maximum a posteriori (MAP) model. Each of the five candidate models is evaluated as the data-generating model in separate experiments, with $n = 100$ simulations per model.

5.1 Results and Interpretation

The main goal of this simulation study is to determine whether the model comparison framework can correctly identify the true data-generating model as the most likely model. Overall, the results show that the correct simulation model is consistently favored, regardless of prior choice or simulation setting. We focus on the results in Table VI, which presents posterior model probabilities for the scenario with $R_0 = 2.0$ using the original priors. Each cell in the table gives the average posterior model probability (and 95% credible intervals) across the 100 simulations for every combination of simulated and fitted model. The results show that the highest posterior model probability (MAP) consistently corresponds to the true data-generating model as intended. Specifically;

- **Baseline model**: correctly identified 99% of the time; mean posterior probability = 0.94 (CI: [0.77, 0.99]).
- **SSE model**: identified 93% of the time; mean = 0.90 (CI: [0.43, 1.00]).
- **SSI model**: identified 67% of the time; mean = 0.63 (CI: [0.06, 1.00]).
- **SSEB model**: identified 97% of the time; mean = 0.90 (CI: [0.68, 1.00]).
- **SSIB model**: identified 63% of the time; mean = 0.55 (CI: [0.05, 0.99]).

Although the SSI and SSIB models show slightly lower identification rates, they are still the most probable models in the majority of simulations involving their own generated data. Another key pattern is that super-spreading events (SSE/SSEB) and super-spreading individuals (SSI/SSIB) models often identify each other as the second most probable model. These finding support our decision to model super-spreading via distinct mechanisms— super-spreading events versus super-spreading individuals. These findings suggest our framework can reliably identify the correct underlying model of epidemic transmission and provides confidence in its application to real-world data. We next examine the results of each model analyses individually.

	Posterior Probabilities of Fitted Models. $R_0 = 2$, Num days = 50					
		**Baseline**	**SSE**	**SSI**	**SSEB**	**SSIB**
**Simulation Model**	**% MAP**					
Baseline	**99**	**0.94 [0.77, 0.99]**	0.001	0.024	0.034	0.001
SSE	**93**	0	**0.90 [0.43, 1.0]**	0	0.10	0
SSI	**67**	0.09	0.02	**0.63 [0.06, 1.0]**	0.10	0.152
SSEB	**97**	0.002	0.101	0	**0.90 [0.68, 1.0]**	0.001
SSIB	**63**	0.19	0	0.232	0.023	**0.55 [0.05, 0.99]**

Table VI: Summary of estimated posterior model probabilities (mean, CI) for each combination of simulated model and fitted model for $N = 100$ simulations from each model in the left hand column . The Baseline model is correctly identified as the most probable model 99% of the time, the SSE model 93% of the time, the SSI model 67% of the time, the SSEB model 97% of the time, and the SSIB model 63% of the time. These results indicate high sensitivity for the Baseline, SSE, and SSEB models, with notable uncertainty for the SSI and SSIB models.

5.1.1 Baseline Simulation Model

For analyses of the Baseline model we simulate 100 different epidemic time-series datasets from the Baseline model, $\boldsymbol{I}_{\{[1:\text{T}],\ \text{Baseline}\}}$, for $R_0 = 2$. We calculate the model evidence of each of the five models when fit to this data and in turn we calculate the posterior model probability $P(M_i|\boldsymbol{I}_{\{[1:\text{T}],\ \text{Baseline}\}})$ of each model. The results are displayed in Figure II. For 99 out of the 100 simulations, the Baseline model is correctly identified as the most likely candidate model with a mean posterior model probability of 0.94 (CI: [0.77, 0.99]) when compared against the four other models. The results demonstrate a high degree of sensitivity for the Baseline model.

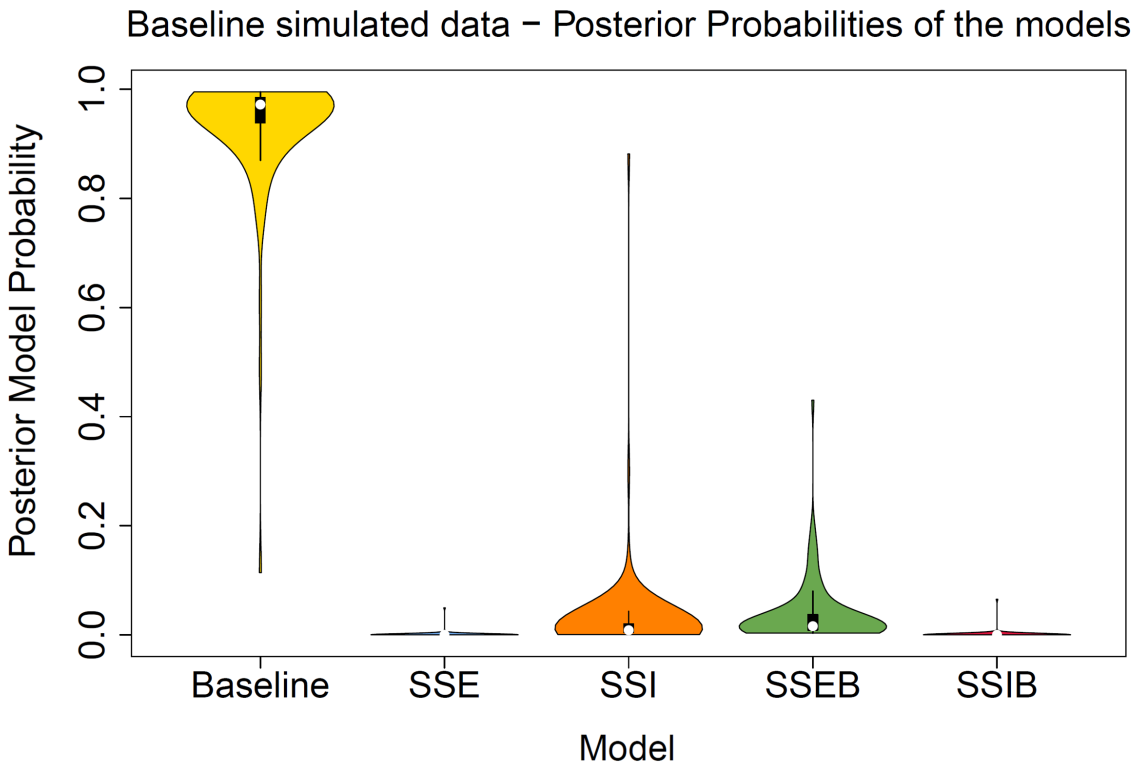

Figure II: Results of our model selection simulation study when applied to multiple simulations from the Baseline model ($N = 100$). Each simulated epidemic is of length $T = 50$ and $R_0 = 2$. The posterior model probability of each of the five models is calculated when fit to the simulated data. For 99 of the 100 simulations, the Baseline model is correctly identified as the most likely candidate model, with a mean posterior model probability of 0.94 (CI: [0.77, 0.99]).

5.1.2 SSE Simulation Model

For analyses of the SSE model, we simulate 100 different epidemic time-series from the SSE model, $\boldsymbol{I}_{\{[1:T],\ \mathrm{SSE}\}}$, for $R_0 = 2, k = 0.2$. We calculate the model evidence of each of the five models when fit to the data and in turn the posterior model probability $P(M_i|\boldsymbol{I}_{\{[1:T],\ \mathrm{SSE}\}})$ of each model. The results are displayed in Figure III. For 93 of the 100 runs, the SSE model is correctly identified as the most likely candidate model with a mean posterior model probability of 0.90 (CI: [0.43, 1.0]) when compared against the four other models. Interestingly, the SSEB model is the second most likely model when fit to SSE data with a mean posterior probability of 0.10. This outcome aligns with our expectation as both models are super-spreading events models. The results indicate a high degree of sensitivity for the SSE model.

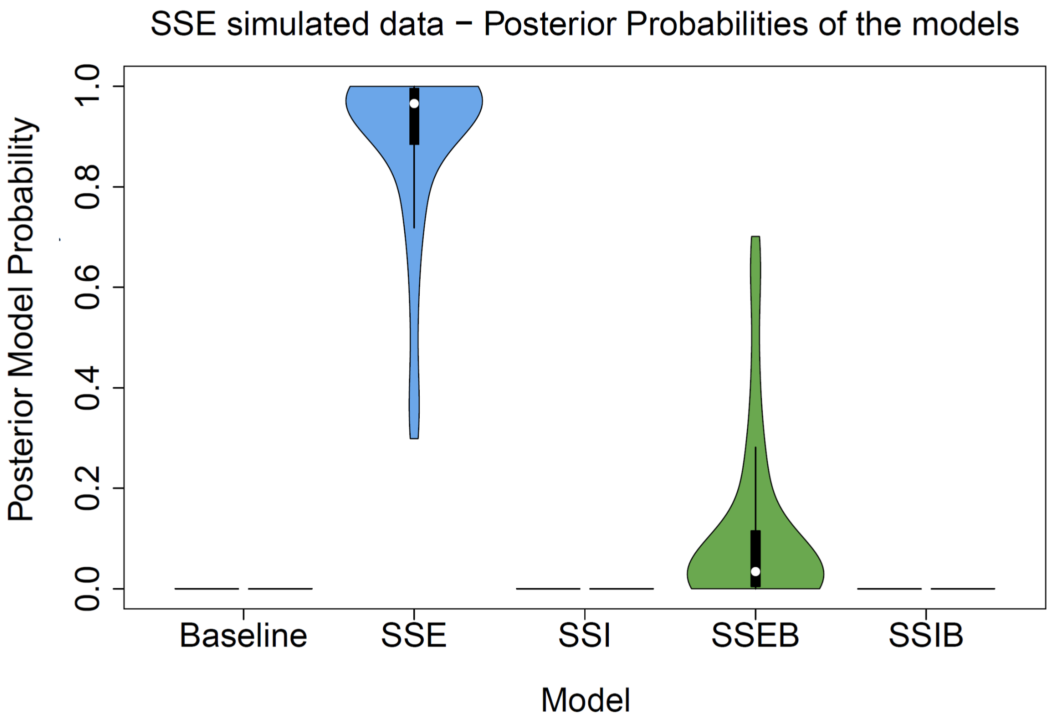

Figure III: Results of our model selection simulation study when applied to multiple simulations from the SSE model ($N = 100$). The posterior model probability of each of the five models is calculated when fit to SSE data. For 93 of the 100 simulations, the SSE model is correctly identified as the most likely candidate model with a mean posterior model probability of 0.90 (CI: [0.43, 1.0]) when compared against the four other models.

5.1.3 SSI Simulation Model

For analyses of the SSI model, we simulate 100 different epidemic time-series from the SSI model, $\boldsymbol{I}_{\{[1:T],\,SSI\}}$, for $R_0 = 2, k = 0.2$. We calculate the model evidence of each of the five models when fit to the data and in turn the posterior model probability $P(M_i|\boldsymbol{I}_{\{[1:T],\,SSI\}})$ of each model. The results are displayed in Figure IV. For 67 of the 100 runs, the SSI model is correctly identified as the most likely candidate model with a mean posterior model probability of 0.63 (CI: [0.05, 1.0]) when compared against the four other models. Although the SSI model emerges as the MAP most frequently among all models, it carries a higher level of uncertainty compared to the preceding models. As previously discussed, inherent uncertainty exists in the inference of the individual models. The additional augmented parameters, $\boldsymbol{\nu^+}_{[1]} = [\nu_1^+, \nu_2^+, \ldots, \nu_t^+, \ldots, \nu_T^+]$ which we need to infer contribute to this. Nevertheless, our model comparison approach successfully identifies the SSIB model, another super-spreading individuals model, as the second most likely model when fit to the SSI data, with a mean posterior probability of 0.152. This result aligns with our expectations since both models are designed to account for super-spreading infections.

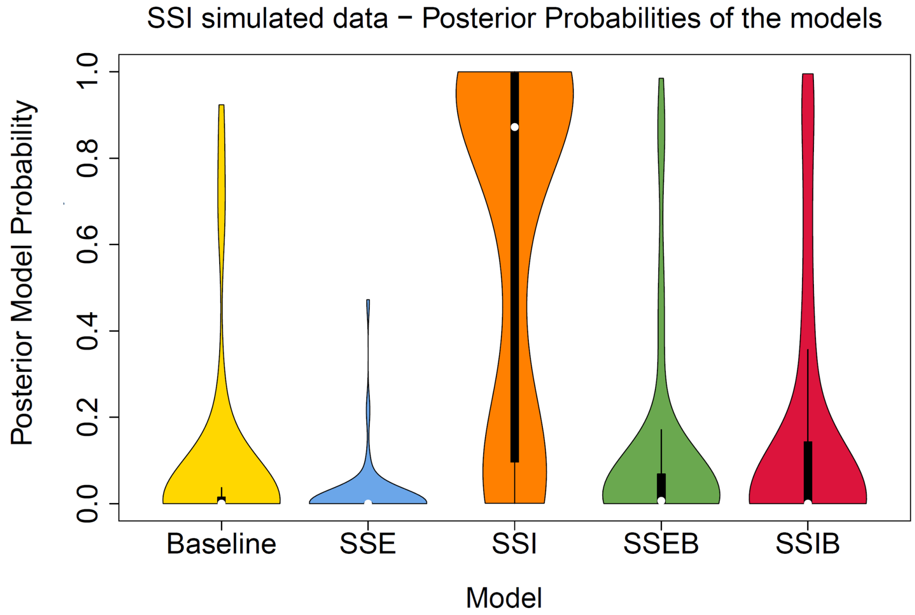

Figure IV: Results of our model selection simulation study when applied to multiple simulations from the SSI model ($N = 100$). Each simulated epidemic is of length $T = 50$ and $R_0 = 2, k = 0.2$. The posterior model probability of each of the five models is calculated when fit to the simulated data. For 67 of the 100 simulations, the SSI model is correctly identified as the most likely candidate model with a mean posterior model probability of 0.63 (CI: [0.05, 1.0]) when compared against the four other models. Although the SSI model emerges as the MAP most frequently among all models there is inherent uncertainty in the inference of the individual models.

5.1.4 SSEB Simulation Model

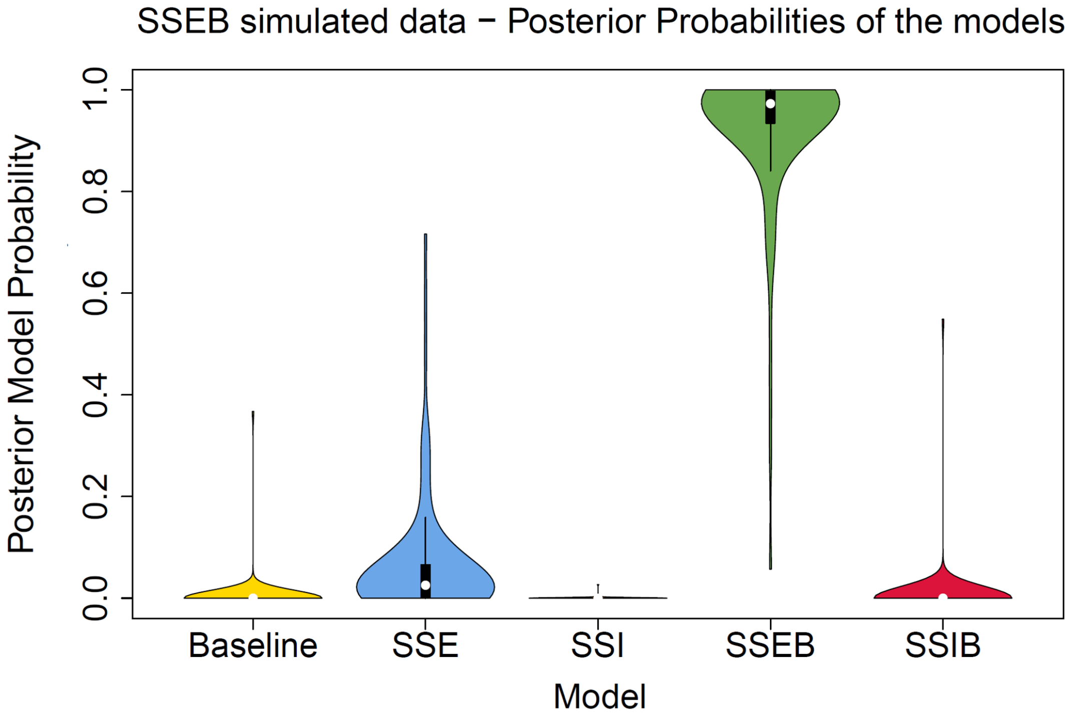

Figure V: Results of our model selection simulation study when applied to multiple simulations from the SSEB model ($N = 100$). The posterior model probability of each of the five models is calculated when fit to the simulated data. For 97 of the 100 simulations, the SSE model is correctly identified as the most likely candidate model with a mean posterior model probability of 0.0896 (CI: [0.68, 1.0]) when compared against the 4 other models.

5.1.5 SSIB Simulation Model

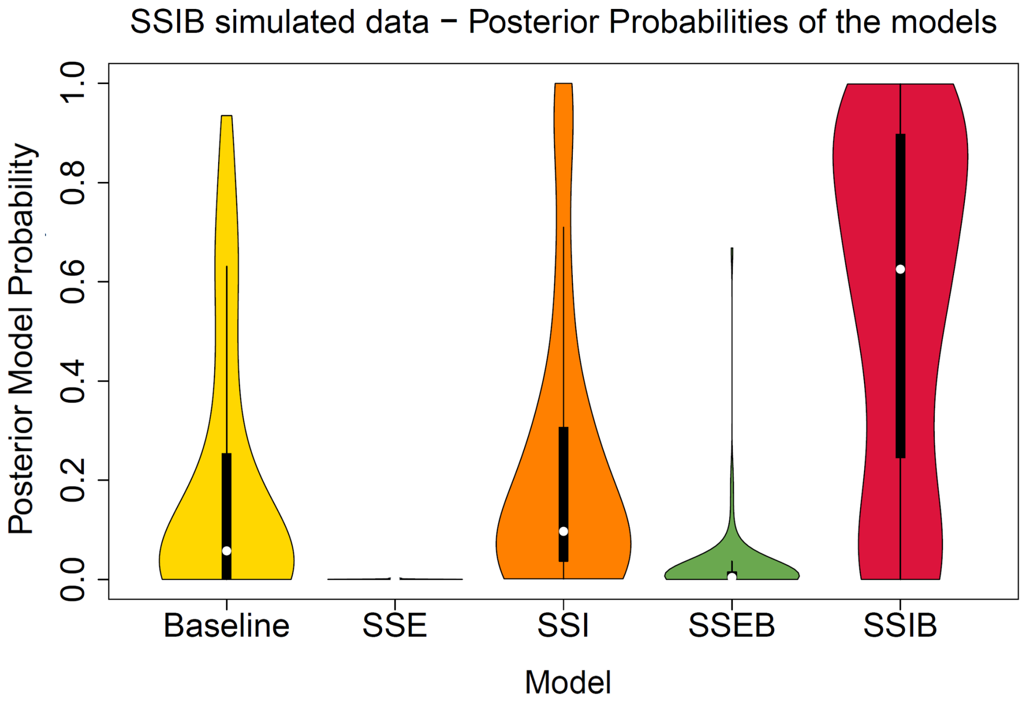

Figure VI: Results of our Model Comparison Framework applied to multiple simulations (N = 100) from the SSIB model. The posterior model probability of each of the five models is calculated when fit to the simulated data. For the majority of the $N = 100$ runs, the SSIB model is correctly identified as the most likely candidate model, as its' posterior model weight is closest to 1 (mean; 0.55, 95 % Credible interval; [0.01, 0.99])

References

Adam, D., Gostic, K., Tsang, T., Wu, P., Lim, W. W., Yeung, A., Wong, J., Lau, E., Du, Z., Chen, D., Ho, L.-M., Martín-Sánchez, M., Cauchemez, S., Cobey, S., Leung, G., and Cowling, B. (2022). Time-varying transmission heterogeneity of SARS and COVID-19 in Hong Kong. *Research Square*.

Biggerstaff, M., Cauchemez, S., Reed, C., Gambhir, M., and Finelli, L. (2014). Estimates of the reproduction number for seasonal, pandemic, and zoonotic influenza: a systematic review of the literature. *BMC infectious diseases*, 14(1):1–20.

Cori, A., Ferguson, N. M., Fraser, C., and Cauchemez, S. (2013). A new framework and software to estimate time-varying reproduction numbers during epidemics. *American journal of epidemiology*, 178(9):1505–1512.

Craddock, H. (2024). Super-spreading in epidemics: A bayesian modelling framework with multi-model comparison. Submitted for the degree of Doctor of Philosophy. Available at: `https://wrap.warwick.ac.uk/id/eprint/189371/`.

Delamater, P. L., Street, E. J., Leslie, T. F., Yang, Y. T., and Jacobsen, K. H. (2019). Complexity of the basic reproduction number (r0). *Emerging infectious diseases*, 25(1):1.

Endo, A., Abbott, S., Kucharski, A. J., Funk, S., et al. (2020). Estimating the overdispersion in COVID-19 transmission using outbreak sizes outside China. *Wellcome open research*, 5.

Grant, R., Nguyen, L.-B. L., and Breban, R. (2020). Modelling human-to-human transmission of monkeypox. *Bulletin of the World Health Organization*, 98(9):638.

Ho, F., Parag, K. V., Adam, D. C., Lau, E. H., Cowling, B. J., and Tsang, T. K. (2023). Accounting for the potential of overdispersion in estimation of the time-varying reproduction number. *Epidemiology*, 34(2):201–205.

James, A., Plank, M. J., Hendy, S., Binny, R. N., Lustig, A., and Steyn, N. (2021). Model-free estimation of COVID-19 transmission dynamics from a complete outbreak. *PLoS One*, 16(3):e0238800.

Johnson, V. E. and Rossell, D. (2010). On the use of non-local prior densities in bayesian hypothesis tests. *Journal of the Royal Statistical Society Series B: Statistical Methodology*, 72(2):143–170.

Kim, W.-H. and Cho, S. (2021). Estimation of the basic reproduction numbers of the subtypes h5n1, h5n8, and h5n6 during the highly pathogenic avian influenza epidemic spread between farms. *Frontiers in Veterinary Science*, 8:597630.

Kucharski, A. and Althaus, C. L. (2015). The role of superspreading in Middle East respiratory syndrome coronavirus (MERS-CoV) transmission. *Eurosurveillance*, 20(25):21167.

Liu, Y., Gayle, A. A., Wilder-Smith, A., and Rocklöv, J. (2020). The reproductive number of covid-19 is higher compared to sars coronavirus. *Journal of travel medicine*.

Riley, S., Fraser, C., Donnelly, C. A., Ghani, A. C., Abu-Raddad, L. J., Hedley, A. J., Leung, G. M., Ho, L.-M., Lam, T.-H., Thach, T. Q., et al. (2003). Transmission dynamics of the etiological agent of sars in hong kong: impact of public health interventions. *Science*, 300(5627):1961–1966.

Wallinga, J. and Teunis, P. (2004). Different epidemic curves for severe acute respiratory syndrome reveal similar impacts of control measures. *American Journal of Epidemiology*, 160(6):509–516.

Wang, L., Didelot, X., Yang, J., Wong, G., Shi, Y., Liu, W., Gao, G. F., and Bi, Y. (2020). Inference of person-to-person transmission of COVID-19 reveals hidden super-spreading events during the early outbreak phase. *Nature communications*, 11(1):5006.

Wong, Z., Bui, C., Chughtai, A., and Macintyre, C. (2017). A systematic review of early modelling studies of ebola virus disease in west africa. *Epidemiology & Infection*, 145(6):1069–1094.
